# Wild Geese Migration Optimization Algorithm: A New Meta-Heuristic Algorithm for Solving Inverse Kinematics of Robot

**DOI:** 10.1155/2022/5191758

**Published:** 2022-09-27

**Authors:** Honggang Wu, Xinming Zhang, Linsen Song, Yufei Zhang, Lidong Gu, Xiaonan Zhao

**Affiliations:** ^1^School of Mechanical and Electrical Engineering, Changchun University of Science and Technology, Changchun 130022, China; ^2^School of Mechatronic Engineering and Automation, Foshan University, Foshan 528225, China; ^3^School of Computer Science and Technology, Changchun University of Science and Technology, Changchun 130022, China

## Abstract

This paper proposes a new meta-heuristic algorithm, named wild geese migration optimization (GMO) algorithm. It is inspired by the social behavior of wild geese swarming in nature. They maintain a special formation for long-distance migration in small groups for survival and reproduction. The mathematical model is established based on these social behaviors to solve optimization problems. Meanwhile, the performance of the GMO algorithm is tested on the stable benchmark function of CEC2017, and its potential for dealing with practical problems is studied in five engineering design problems and the inverse kinematics solution of robot. The test results show that the GMO algorithm has excellent computational performance compared to other algorithms. The practical application results show that the GMO algorithm has strong applicability, more accurate optimization results, and more competitiveness in challenging problems with unknown search space, compared with well-known algorithms in the literature. The proposal of GMO algorithm enriches the team of swarm intelligence optimization algorithms and also provides a new solution for solving engineering design problems and inverse kinematics of robots.

## 1. Introduction

The rapid development of informational and intelligent technology has spawned many new intelligent application requirements. It has also led to many new optimization problems with nonlinearity, complexity, and constraints in engineering, science, economics, management, and other fields. Traditional optimization methods have been unable to meet the needs of computing, and seeking efficient optimization algorithms has become a research hotspot in related disciplines [1–3]. The meta-heuristic algorithms are widely used to solve optimization problems due to the advantages of simplicity, flexibility, and derivation-free mechanism [4–6]. The algorithm is based on mathematics and finds the best possible solution from all candidate solutions through an iterative calculation mechanism [7, 8].

Most of the meta-heuristic algorithms are inspired by the social nature of biological swarms, the laws of natural phenomena, and human intelligence. In general, the algorithms are mainly divided into three categories. The algorithms based on the laws of natural phenomena can be divided into evolutionary laws and physical laws. The evolution-based algorithms mainly include genetic algorithm (GA) [9], differential evolution algorithm (DE) [10], black hole algorithm (BH) [11], natural aggregation algorithm (NAA) [12], barnacles mating optimizer (BMO) [13], biogeography-based optimization (BBO) [14], bird mating optimizer (BMO) [15], and so on. Among them, GA algorithm is inspired by Darwin's theory of evolution. Each individual in the algorithm is assigned a specific gene, and the iterative optimization process is achieved by the genetic evolution of individual genes. NAA algorithm is inspired by the collective decision making intelligence of the group-living animals. Individuals will make decisions about entering/leaving a subpopulation by the quality and crowding of the subpopulation to achieve localization and generalization search for the problem space. The physics-based algorithms mainly include simulated annealing algorithm (SA) [16], central force optimization algorithm (CFO) [17], electromagnetic field optimization algorithm (EFO) [18], water evaporation optimization algorithm (WEO) [19], gravitational search algorithm (GSA) [20], and so on. The algorithms based on human social behavior mainly include teaching-learning-based optimization algorithm (TLBO) [21], student psychology-based optimization algorithm (SPBO) [22], social-based algorithm (SBA) [23], and so on. The kho-kho optimization (KKO) algorithm [24] and battle royale algorithm (BRO) [25] are inspired by players' rules in the games.

At present, the most studied algorithm is based on biological swarm behavior, which is also called swarm intelligence optimization algorithm. The algorithms mainly include particle swarm optimization algorithm (PSO) [26], bat-inspired algorithm (BA) [27], artificial bee colony algorithm (ABC) [28], fruit fly optimization algorithm (FOA) [29], migrating birds optimization (MBO) [30], cuckoo search algorithm (CS) [31], cuttlefish algorithm (CFA) [32], ant colony optimization algorithm (ACO) [33], moth-flame optimization algorithm (MFO) [34], mayfly optimization algorithm (MA) [35], chicken swarm optimization algorithm (CSO) [36], naked mole-rat algorithm (NMR) [37], and so on. Among them, the PSO algorithm is inspired by the social behavior of bird swarm. Each particle continuously explores the solution space in this algorithm to find the global optimum. The position update strategy is based on the historical optimal position and the global optimal position of each particle. The inspiration of the MBO algorithm comes from the V flight formation during the migration of birds. The position update is implemented sequentially from the optimal value, and the position of current individual is compared with its neighbors. If the fitness of the neighbor is better, the current individual will be replaced. The CS algorithm is a meta-heuristic algorithm based on the cuckoo's brood parasitic behavior and the bird's Lévy flight behavior. The algorithm is to search for the global optimal solution through the strategy of Lévy flight and random walk. The CFA algorithm is inspired based on the colour changing behavior of cuttlefish. The population will be divided into four independent groups in the algorithm, and an independent search strategy is designed for each group by simulating the two processes of reflection and visibility.

The meta-heuristic algorithm is proposed not only for theoretical research in the laboratory, but more importantly, it is hoped to achieve satisfactory results in different practical application fields. The research of many algorithms is based on specific practical applications and explores their excellent computational performance. For instance, Taymaz proposed the BRO algorithm [25] and applied it to solve the inverse kinematics problem of the PUMA560 robot. The research shows that the BRO algorithm achieves excellent results in the position solution. Amir et al. proposed the CS algorithm [31] and verified its excellent performance through 13 engineering design problems. Seyedali proposed the ant lion optimizer (ALO) [38] and applied it to the design of ship propellers. The smooth blade shape is found through the ALO algorithm to improve the propeller efficiency. Mirjalili et al. proposed the grey wolf algorithm (GWO) [39] and applied it to optimize the BSPCW structure in the optical buffer design problem. The optimized structure has a good bandwidth and does not require any frequency mixing. Seyedali proposed the sine cosine algorithm (SCA) [40] and applied it to the two-dimensional design of aircraft wings. Minimal drag is the goal of structural optimization. The optimization results show that the drag is reduced from 0.009 to 0.0061, and the effect is pronounced. Li et al. proposed the slime mold algorithm (SMA) [41] and verified the algorithm's performance on multiple benchmark functions and five practical engineering design problems. The SMA algorithm exhibits satisfactory computational performance in solving engineering problems. Kaur et al. proposed the tunicate swarm algorithm (TSA) [42] and applied it to the solution of constrained and unconstrained engineering problems. The applicability of the TSA algorithm is verified.

In order to mimic nature more effectively and improve the search performance of the algorithm [43], fitness-distance balance (FDB) proposed by Kahraman et al. [44] has made significant contributions, which combines FDB with the symbiotic organisms search algorithm (FDB-SOS). Compared with 13 meta-heuristic search (MHS) techniques, the excellent performance of the FDB-SOS algorithm is verified on 90 benchmark functions. Aras et al. [45] proposed an FDBSFS algorithm, which uses the FDB mechanism to optimize the stochastic fractal search algorithm. Compared with 39 MHS algorithms, it verifies the powerful search performance and the competitiveness of the FDBSFS algorithm, on 89 unconstrained benchmark functions and 5 constrained engineering problems. Ozkaya et al. [46] redesigned the mutation operator of the improved adaptive differential evolution (LSHADE) algorithm by the FDB mechanism, which is defined as the FDB-LSHADE algorithm. Compared with other 8 MHS algorithms, the FDB-LSHADE algorithm shows excellent performance on CEC14, CEC17, and energy hub economic dispatch problems. To achieve higher performance goals, the application range is wider. The researchers consider combining swarm intelligence algorithms with other deep learning methods. For instance, Ghasemi-Darehnaei et al. [47] proposed a swarm intelligence ensemble deep transfer learning method (SI-EDTL) and used the whale optimization algorithm (WOA) to select the optimal hyperparameters of SI-EDTL. Meanwhile, SI-EDTL is applied to multiple vehicle detection in unmanned aerial vehicle (UAV) images. Basha et al. [48] proposed an improved Harris hawks optimization algorithm to optimize the convolutional neural network (CNN) architecture. Compared with other similar methods, the network achieves superior performance in classifying various grades of brain tumors. Singh et al. [49] proposed a multistage particle swarm optimization (MPSO) algorithm to explore the CNN architecture and its hyperparameters (MPSO-CNN), which achieved better performance on 5 benchmark datasets. Hilal et al. [50] studied a remote sensing image classification model (FCMBS-RSIC) based on fuzzy logic and bird swarm algorithm and performed performance verification on benchmark open-access datasets. The FCMBS-RSIC model has enhanced results compared to other state-of-the-art methods. Zivkovic et al. [51] proposed a framework to improve the prediction accuracy of COVID-19 cases, which is an adaptive neuro-fuzzy inference system trained by an improved beetle antenna search algorithm. Kumar and Jaiswal [52] proposed a cognitive-driven analytics model (CNN-WSADT) for real-time data classification. It combines three deep learning methods of CNN, wolf-search algorithm, and decision tree.

With the efforts of the researchers, new meta-heuristic algorithms are proposed every year and applied to solve complex optimization problems in different fields. Each algorithm balances its exploitation and exploration process by setting up a unique search mechanism, which may be intrinsic to the success of the new algorithm [53–55]. However, no single meta-heuristic algorithm satisfies all optimization problems, as explained by the no-free-lunch theorem [56]. In other words, the same algorithm may achieve satisfactory results on one optimization problem but may exhibit poor computational performance on another. Therefore, with the continuous innovation of science and technology, the complexity and challenge of optimization problems continue to increase. While improving traditional algorithms, researchers also need to propose new algorithms and theories. This motivates us to propose a new meta-heuristic algorithm, inspired by wild geese migration. There is no prior study on this topic in the optimization algorithm literature to the authors' knowledge.

This paper describes a new meta-heuristic optimization algorithm (GMO). The algorithm simulates the social behavior of wild geese migration and designs multiple migration groups. The iterative process of the GMO algorithm mainly refers to the behavior of randomly establishing migration groups, synchronous migration, and free foraging. The random establishment of the migration group in the algorithm is that its members are randomly generated with the head goose (the best individual in the migration group) as the center. The synchronous migration means that individuals in each migration group update their positions in equal steps. The free foraging refers to individuals moving within a small random range. To evaluate the performance of the GMO algorithm, the simulation experiments are carried out by 29 stable benchmark functions in CEC2017. At the same time, the algorithm is applied to solve five engineering design problems and the inverse kinematics problem of 7R 6DOF robot and is compared with other algorithms reported in the literature. The results show that the computational performance of the GMO algorithm is more competitive, and it effectively solves practical engineering problems.

The main contributions of this paper are as follows:

1. The development and latest research results of meta-heuristic algorithms are analyzed through literature, which provides more theoretical basis and reference value for the new algorithm proposed in this paper.
2. This paper proposes a new swarm intelligence algorithm, named GMO algorithm, which is inspired by the social behavior of long-distance migration of wild geese swarm. In the algorithm, the search mechanism of randomly establishing migration groups, synchronous migration, and free foraging is designed, which effectively balanced the exploitation and exploration process in the search space.
3. Simulation experiments are carried out in the 29 stable benchmark functions of CEC2017, and each function is tested on 10, 30, 50, and 100 dimensions. The experimental results of GMO algorithm and 5 other algorithms are compared in detail. It is shown that the GMO algorithm has good convergence accuracy and speed, strong stability, and short running time.
4. The GMO algorithm is applied to five engineering design problems in this paper. Compared with the results reported in other studies, the GMO algorithm has shown good results in the face of practical problems in different search spaces. The applicability and feasibility of the algorithm to solve engineering optimization problems are verified.
5. The GMO algorithm is used to solve the inverse kinematics problem of the 7R 6DOF robot. The results show that the GMO algorithm is better than other comparative algorithms in the solution of the inverse kinematic pose problem and has a higher solution accuracy. The algorithm provides a new method for solving the inverse kinematics problem of the robot.

The rest of this paper is organized as follows.  Section 2 presents the GMO algorithm and introduces its primary sources of inspiration and design principles.  Section 3 gives the simulation experiment of the GMO algorithm by benchmark functions, comparing it with other algorithms to verify its computational performance.  Section 4 is devoted to solving five engineering optimization problems using the GMO algorithm and proving the algorithm's applicability.  Section 5 successfully solves the inverse kinematics problem of the 7R 6DOF robot through the GMO algorithm. Finally, the conclusion of this paper and directions for possible future research are given in Section 6.

## 2. GMO Algorithm

In this section, the inspiration for the GMO algorithm is first introduced to better understand the proposed methodology. Then, the mathematical model of the algorithm is provided, and its implementation flow and pseudocode are described. Finally, the time complexity analysis of the GMO algorithm is carried out.

### 2.1. Inspiration

**Figure 1:**
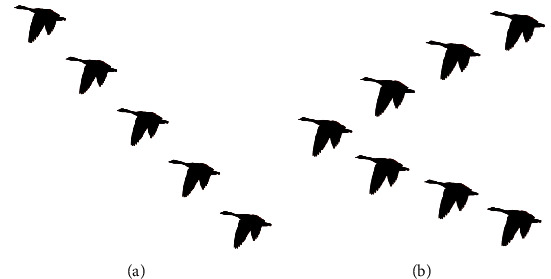
Flight formation of wild geese. (a) Line-shaped arrangement. (b) V-shaped arrangement.

The wild goose is a general term for birds of the genus goose, and it is also an excellent air traveller. Every autumn, they fly in droves from Siberia to the south for the winter. The following spring, they will return to Siberia to lay eggs and breed after a long journey. In the migration process, each migration group consists of many geese, and the experienced head geese lead them to fly in line-shaped or V-shaped arrangement, as shown in Figure 1. This is a miraculous natural phenomenon.

During the flight of the wild geese, wild geese generate vortices and updrafts by constantly flapping their wings. The wild geese that follow closely will fly in these air currents, saving a lot of energy. However, the head geese have no available updraft resources, and their physical energy will be consumed the fastest. Therefore, to ensure the continuity of the air flight, each wild geese migration group needs to change formation and head geese frequently on long-distance flights. Meanwhile, the wild geese group migration is also conducive to exchanging information and avoiding natural enemies [57].

### 2.2. Algorithm Principles and Mathematical Models

The GMO algorithm's initial population is randomly generated in the solution space, and a certain number of wild geese are selected as the initial head geese. The wild geese swarm migrate under the leadership of the head geese. The population size of the wild geese in the GMO algorithm is *N*, and the number of the head geese is *M*. The migration group initial radius size is set to *L* (*L*=*u* *d* − *l* *d*/*N*).

#### 2.2.1. Formation of Migration Groups

In each iteration process, the migration groups are reestablished according to the position of the head geese. The members of each group are randomly distributed within the radius *L* with the head goose as the center. Its purpose is to realize the replacement of the head geese and the transformation of the formation. The mathematical model is as follows:

(1) $$\begin{array}{c} \left\{\begin{array}{cc} x_{i}^{t}=x_{j}^{t}\text{,} & ifi=b*\left(j-1\right)+1\text{,}\\ x_{i}^{t}=x_{j}^{t}-L+2L*ran\;d\left(1,di\;m\right)\text{,} & else\text{,} \end{array}\right. \end{array}$$

where *x*_*i*_^*t*^ represents the position of the *i*-th individual at the *t*-th iteration (*i* = 1, 2,…, *N*). *T* is the maximum number of iterations (*t* = 1, 2,…, *T*). *x*_*j*_^*t*^ represents the position of the *j*-th head goose individual at the *t*-th iteration (*j* = 1, 2,…, *M*). *b* represents the number of migration groups (*b* *=* *N/M*).

#### 2.2.2. Synchronized Flight

**Figure 2:**
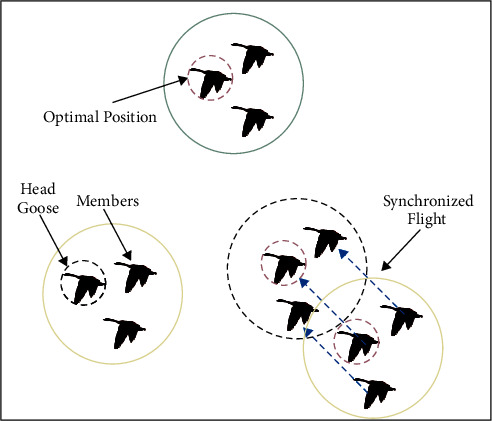
The flight process of a migration group.

During the migration process of the wild geese, the head geese in nature mainly rely on environmental information, historical memory, and flight experience to guide the migration. Meanwhile, each migration group member maintains a relatively fixed position to fly with the head goose. The synchronous flight strategy is used in the GMO algorithm to simulate the flight characteristics of wild geese, and the flight steps in the migration group members are set to be equal. The individuals' position update information in the migration group is derived from the head goose, which is mainly based on the optimal position and refers to the position information of other head goose. The schematic diagram of the flight process of a migration group is shown in Figure 2, and the mathematical model is as follows:

(2) $$\begin{array}{c} x_{i}^{t+1}=x_{i}^{t}+c_{1}\left(x_{best}^{t}-x_{j}^{t}\right)+c_{2}\left(x_{k}^{t}-x_{j}^{t}\right) \end{array}$$

where *x*_best_^*t*^ represents the global optimal individual and *x*_*k*_^*t*^ is the randomly selected head goose individual. *x*_*i*_^*t*^ and *x*_*j*_^*t*^ represent the members and the head goose in a migration group, respectively. The flight step size *c*_1_ ∈ [0, 1], and *c*_2_ is calculated by

(3) $$\begin{array}{c} \left\{\begin{array}{cc} c_{2}=\exp\frac{fit\left(j\right)-{fit}_{ave}}{{fit}_{worse}-{fit}_{best}}\text{,} & fit\left(j\right)\leq {fit}_{ave}\text{,}\\ c_{2}=\exp\frac{fit\left(j\right)+{fit}_{ave}-2{fit}_{best}}{{fit}_{worse}-{fit}_{best}}\text{,} & fit\left(j\right)>{fit}_{ave}\text{,} \end{array}\right. \end{array}$$

where fit(*j*) is the fitness value of the head goose, fit_worse_, fit_ave_, and fit_best_ represent the worst, average, and best fitness value of the head geese, respectively, and *c*_2_ is mainly used to control the proportion of other head geese's experience information. If fit(*j*) ≤ fit_ave_, it indicates that the value of fit(*j*) is small and means that *x*_*j*_^*t*^ is an excellent head goose and does not need to learn more information from other head goose. The exact opposite is true when fit(*j*) > fit_ave_.

#### 2.2.3. Free Foraging

**Figure 3:**
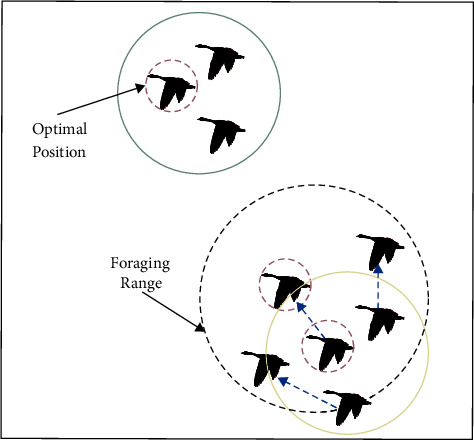
The foraging process of migratory group members.

Resting and foraging are inevitable for migratory groups during long-distance flights. Wild geese often choose lakes or larger bodies of water in nature as the foraging area. During the free foraging process, the migration group members will randomly explore according to the information of the head goose and maintain a certain connection in a small area. At the same time, the migration group maintains the movement trend by the optimal location information. After finishing foraging, the wild geese will regroup and migrate. A schematic diagram depicting the free foraging process is shown in Figure 3, and the mathematical model is as follows:

(4) $$\begin{array}{c} x_{i}^{t+1}=x_{i}^{t}+c_{3}\left(x_{j}^{t}-x_{i}^{t}+L\right)+c_{4}\left(x_{best}^{t}-x_{j}^{t}\right), \end{array}$$

where *c*_3_ and *c*_4_ are random numbers between [0, 1], respectively, used to control the movement step size of individuals during the foraging process. *L* is the radius of the group range, which is used to control the distance between the migration group members and the head goose.

#### 2.2.4. Selection of the Head Geese

During the long-distance migration of wild geese, the head geese are the most crucial individuals, and they are the leaders of the entire wild geese swarm. The head geese must be replaced frequently to achieve high flight durability. Therefore, the optimal individuals in each migration group will be selected as the head geese of the new generation after each location update of the GMO algorithm. This selection strategy not only allows the head geese to carry excellent location information but also ensures the dispersion of the head geese's positions, so that the algorithm has an excellent ability to balance exploitation and exploration.

After the head geese are all replaced, the migration group radius (*L*) is reduced by equation (5). The purpose is to increase the density of members in the group and improve the exploration accuracy of the algorithm.

(5) $$\begin{array}{c} L=L*\left(1-0.1\left(\frac{t}{T}\right)\right), \end{array}$$

where *T* is the maximum number of iterations and *t* is the current number of iterations.

### 2.3. Implementation of GMO Algorithm

**Figure 4:**
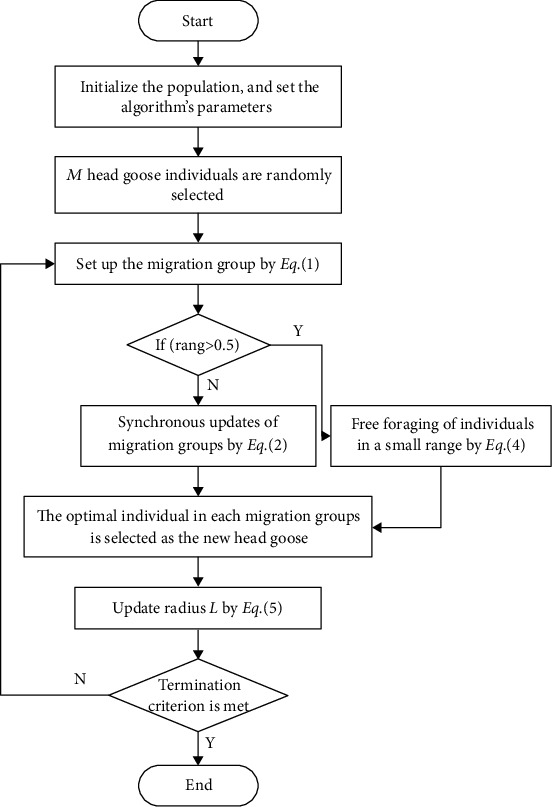
The flowchart of GMO algorithm.

The GMO algorithm is a new stochastic optimization algorithm. Multiple random positions within the solution space are chosen as initial solutions, and then all solutions are iterated and optimized continuously to find the optimal solution. The flowchart and pseudocode of the GMO algorithm are presented in Figure 4 and Algorithm 1, respectively.

### 2.4. Time Complexity

In practical engineering applications, the computational efficiency and computational performance of an algorithm are equally important. The time complexity analysis method is one of the essential means to evaluate the algorithm's efficiency. This method can analyze the algorithm' complexity under the condition that the population number *N* and the number of iterations *T* remain unchanged, and the computational efficiency of the algorithm can be accurately verified. The calculation process of the GMO algorithm mainly includes three parts: population initialization *O*(*N*), the establishment of migration groups *O*(*N* *∗* *T*), and synchronized flight or free foraging *O*(*N* *∗* *T*). Therefore, the time complexity of the GMO algorithm is *O*(GMO) = *O*(*N*) + *O*(*N* *∗* *T*) + *O*(*N* *∗* *T*). The complexity formula has no exponentiation operation and is mainly affected by the basic parameter *N* *∗* *T*. From the above analysis, it can be seen that the GMO algorithm has a lower time complexity.

## 3. Experimental Results and Analyses

### 3.1. Benchmark Functions and Parameter Setting

For a new meta-heuristic algorithm, it is necessary to test the ability in terms of exploitation and exploration through a large amount of quantitative data. In this work, the performance of the GMO algorithm is tested on 29 stable benchmark functions in the CEC2017 technical report (*F*2 function is deprecated in this paper because of its instability) [58]. The specific function names, variable feasible regions, and minimum values are recorded in Table 1, and the detailed function models can be obtained from [58]. In addition, 4 different types of benchmark functions are provided in this table, including unimodal, multimodal, hybrid, and composition functions. The test results of these benchmark functions can infer the potential ability of the GMO algorithm to solve practical problems.

In order to clearly illustrate the excellent computing performance of the GMO algorithm, the five optimization algorithms are selected as the comparison targets, including the PSO, BRO, CSO, ABC, and WOA algorithms. The common parameters of all algorithms are set as follows: the population number *N* = 100, the maximum number of iterations *T* = 500, and the dimension *D* = 10, 30, 50, and 100, and other related parameters are shown in Table 2. Windows 10 operating system is the processing environment for the experimental process, and the PC processor is Inter(R) Core(TM) i5-3470M CPU @3.20 GHz.

### 3.2. Experimental Results

In the calculation process of the meta-heuristic algorithm, the random numbers in the solution space are generally used as the initial values. The calculation result of the algorithm may be different due to the difference in the initial values. Therefore, to avoid the influence of special data on the overall results, 50 independent experiments are performed for each benchmark function, and the same initial values are used for each independent experiment. This section gives the test results data of 6 algorithms on 29 benchmark functions in different dimensions, and the experiment dimensions include *D* = 10, *D* = 30, *D* = 50, and *D* = 100. The specific experimental results are shown in Tables 3 [Table tab4] [Table tab5] [Table tab6] [Table tab7] [Table tab8] [Table tab9] [Table tab10] [Table tab11] [Table tab12] [Table tab13]–14. Among them, the experimental results of unimodal and multimodal benchmark functions in 4 different dimensions are recorded in Tables 3, 6, 9, and 12, respectively. Similarly, the experimental results of the hybrid functions are recorded in Tables 4, 7, 10, and 13, respectively. The experimental results of the composition benchmark functions are recorded in Tables 5, 8, 11, and 14, respectively.

In order to verify the performance of the GMO algorithm, the mean, standard deviation, and running time of each benchmark function in 50 independent experiments are selected as evaluation indicators. Among them, the mean can evaluate the computing power and accuracy of the algorithm, the standard deviation can evaluate the computational stability of the algorithm, and the running time can judge the complexity of the algorithm. In addition, in order to display the experimental results more clearly and intuitively, each table also records the ranking of the average value and the results of the significance test. The rank of average value is determined by the numerical value of the test results. The algorithm with the smallest average value is ranked 1st, and the algorithm with the largest average value is ranked 6th and gives the same rank when the average value is the same but occupies two positions. The final overall ranking of the algorithm is determined by the average of the algorithm's ranking on all functions.

The significance test technique uses statistical methods to explore whether there are significant differences in data distribution. In this paper, the significance test is performed on the 50 calculation results of the GMO algorithm and other comparison algorithms, respectively. The Wilcoxon rank-sum test or the independent sample *t*-test (*T*-test) is used to test the significance of different types of data. According to the data normality test and variance homogeneity test results, the *T*-test is performed for normally distributed data, and Wilcoxon rank-sum test is performed for others. The level of statistical significance is set at *p* = 0.05. *p* < 0.05 means that the calculation result of the GMO algorithm is significantly different from the comparison algorithm, which is recorded as “1” in the table. *p* > 0.05 means negative answer, which is recorded as “0” in the table.

### 3.3. Evaluation of Exploitation and Exploration Capabilities

The unimodal functions (*F*1, *F*3) are often used to verify the exploitation ability of the algorithm because they have only one global optimal value. The multimodal functions (*F*4–*F*10) have an excellent effect on testing the exploration ability of the algorithm because of the characteristics of multiple local optima.

**Figure 5:**
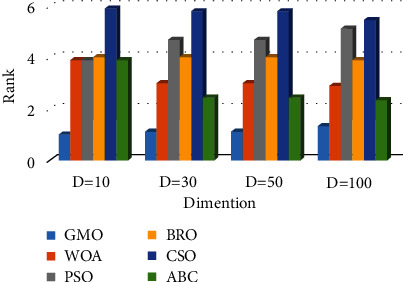
Average ranking of 6 algorithms on unimodal and multimodal functions.

**Figure 6:**
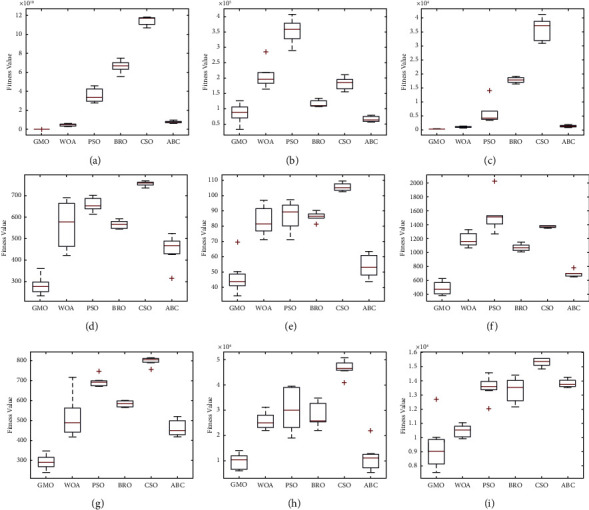
Box plots of the fitness values obtained from 50 experiments for the unimodal and multimodal functions. (a) *F*1. (b) *F*3. (c) *F*4. (d) *F*5. (e) *F*6. (f) *F*7. (g) *F*8. (h) *F*9. (i) *F*10.

The following conclusions can be drawn from the data presented in Tables 3, 6, 9, and 12. In the case of *D* = 10, the calculation results of the GMO algorithm are better than the comparison algorithms. In the case of *D* = 30, the test results of the GMO algorithm on 6 functions are the optimal values, and the test results on the *F*3, *F*6, and *F*9 functions are not the optimal values, but the results are equally competitive. In the case of *D* = 50 and *D* = 100, the test result of the GMO algorithm only on the *F*3 function is not the optimal value. In addition, the comprehensive ranking of the averages in Tables 3, 6, 9, and 12 is shown in Figure 5. It can be seen that the GMO algorithm has the best computation results. Meanwhile, the box plot of the convergence results obtained by 50 experiments on the *F*1–*F*10 functions (taking *D* = 50 as an example) is shown in Figure 6. The figure shows that the GMO algorithm maintains a leading edge in convergence accuracy and stability.

Based on the analysis results of the above data, it can be seen that the GMO algorithm proposed in this paper has good exploitation ability, exploration ability, and computational stability. This may be attributed to two points. One is that the migration group members move randomly in a small area near the head geese during the free foraging process. The other is that the individuals in each migration group keep moving synchronously during the migration process, which effectively expands the scope of exploration.

### 3.4. Ability to Avoid Local Minima

*F*11–*F*20 are hybrid functions, and *F*21–*F*30 are composition functions. These complex functions are obtained by the essential functions' combination, rotation, and offset. The common feature of the functions is that there are a large number of local extrema in the solution space, which makes the solution space closer to the practical problems. The comprehensive ability of the algorithm to balance exploitation and exploration problems can be verified by these functions.

Based on the experimental data provided in Tables 4, 5, 7, 8, 10, 11, 13, and 14, the following conclusions can be drawn.

**Figure 7:**
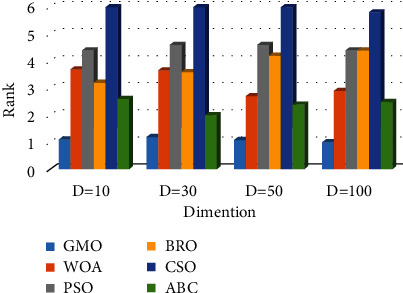
Average ranking on hybrid functions.

**Figure 8:**
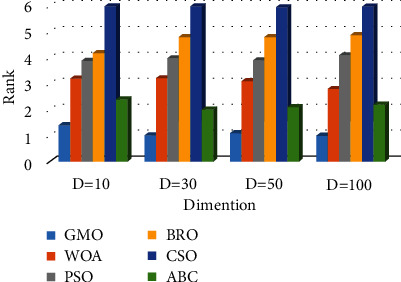
Average ranking on composition functions.

**Figure 9:**
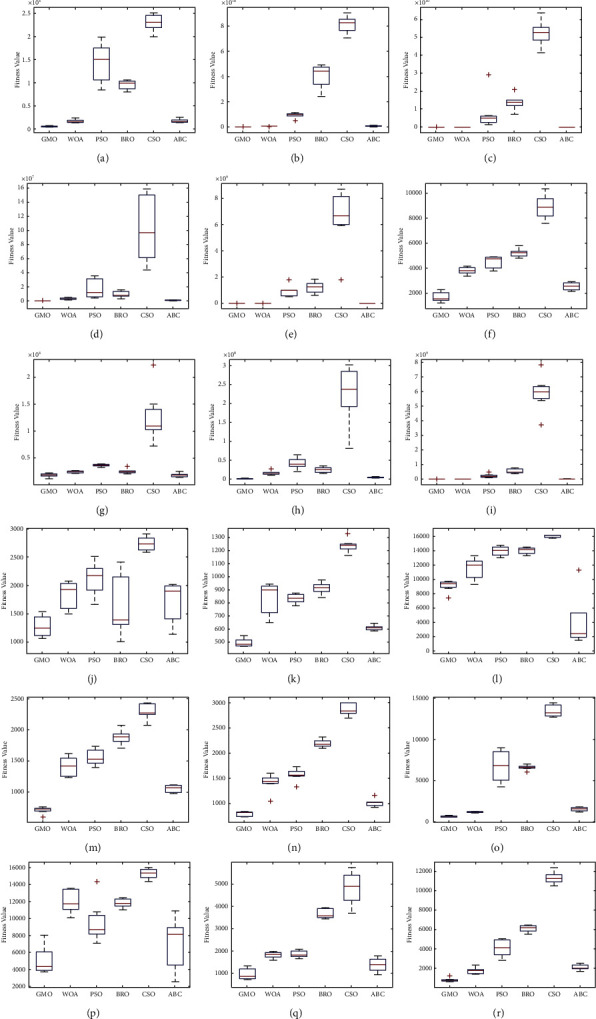
Box plots of the fitness values obtained from 50 experiments for the hybrid and composition functions. (a) *F*11. (b) *F*12. (c) *F*13. (d) *F*14. (e) *F*15. (f) *F*16. (g) *F*17. (h) *F*18. (i) *F*19. (j) *F*20. (k) *F*21. (l) *F*22. (m) *F*23. (n) *F*24. (o) *F*25. (p) *F*26. (q) *F*27. (r) *F*28.

1. In the case of *D* = 10, the GMO algorithm achieves the best calculation results on 17 benchmark functions, and the results only on the *F*17, *F*21, and *F*24 functions are not optimal. In the case of *D* = 30, the GMO algorithm does not achieve the best test results on the *F*15 function, but achieves the best test results on all other benchmark functions. In the case of *D* = 50, the GMO algorithm achieves the best results on 18 hybrid and composition functions, compared with other algorithms. The best experimental results of the other two functions (*F*17, *F*22) are obtained by the ABC algorithm. In the case of *D* = 100, the GMO algorithm obtains the best computational results on all hybrid and composition functions, compared to other algorithms.
2. According to the average of the experimental results, a comprehensive ranking diagram of all algorithms is drawn. The comprehensive ranking of the experiments on the hybrid functions and composition functions is shown in Figures 7 and 8, respectively. The results show that the GMO algorithm ranks first in the solution results of hybrid and composition functions, proving that the GMO algorithm can balance the contradictory problems of exploitation and exploration. The computing power of the GMO algorithm is more competitive compared to other algorithms.
3. The box plots of the convergence results of all algorithms on *F*11–*F*30 functions are shown in Figure 9 (taking *D* = 50 as an example). The figure shows that the GMO algorithm has good stability on hybrid and composition functions.

Based on the above data analysis, the GMO algorithm has the comprehensive ability to solve complex problems of different dimensions. It can well balance the contradiction between exploitation and exploration in the complex solution space, and the algorithm shows good stability. This may be attributed to alternating between synchronous migration and free foraging processes in the GMO algorithm.

### 3.5. Convergence Analysis

**Figure 10:**
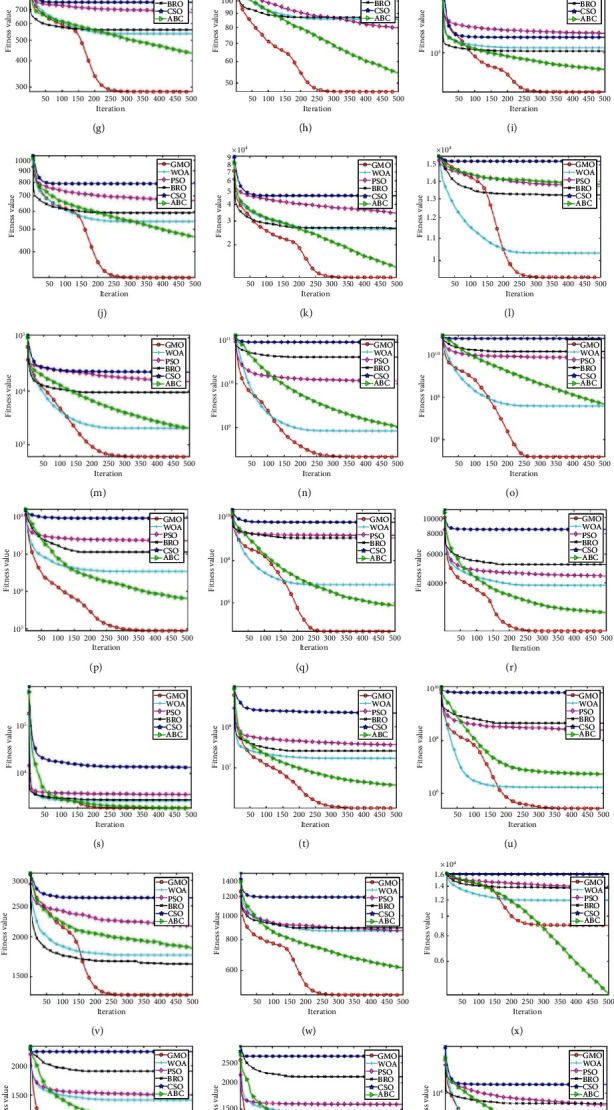
Average convergence curve of 6 algorithms. (a) F1. (b) F3. (c) F4. (d) F5. (e) F6. (f) F7. (g) F8. (h) F9. (i) F10. (j) F11. (k) F12. (l) F13. (m) F14. (n) F15. (o) F16. (p) F17. (q) F18. (r) F19. (s) F20. (t) F21. (u) F22. (v) F23. (w) F24. (x) F25. (y) F26. (z) F27. (aa) F28. (ab) F29. (ac) F30.

The convergence information during the algorithm solving process can be fully displayed in the average convergence curve, which is very important to the computational power of the analysis algorithm. Taking *D* = 50 as an example, this paper gives the average convergence curve of 29 functions by the GMO algorithm and 5 comparison algorithms, as shown in Figure 10. From the overall results, the convergence results of the GMO algorithm are the best on 27 functions and rank second on two functions (F3, F22), which powerfully illustrate the advantage of the GMO algorithm in terms of convergence ability. From the convergence effect of a single function, the convergence speed of the GMO algorithm is slow in the early stage. However, the GMO algorithm converges fast in the middle stage and quickly converges to the global optimum. This may be attributed to the large radius of the migration group in the early stage of the GMO algorithm. The wild geese fully explored the solution space during the synchronous migration process and stored the exploration results. With the continuous iteration of the algorithm, the range radius of the migration group is reduced, and the position of the head geese is continuously optimized, so that the algorithm converges quickly until the best convergence effect is achieved.

### 3.6. Analysis of Significance Test and Running Time

In this section, the experimental results are further analyzed by statistical methods. The significance test (Wilcoxon rank-sum test or *T*-test) results for all data tables in Section 3.2 are counted, as shown in Table 15. In the table, “1” indicates a significant difference between the two samples, and “0” means no significant difference. “+” indicates that the performance of the GMO algorithm is better than other algorithms, and “−” indicates that the performance of the GMO algorithm is worse than other algorithms. Therefore, the number of “1+” in the results is counted, which can strongly demonstrate the advantages of the GMO algorithm.

From the statistical results in Table 15, it can be seen that comparing the GMO algorithm with the WOA, PSO, BRO, and CSO algorithms, there are at least 26 calculation results of “1+,” and comparing the GMO algorithm with the ABC algorithm, there are at least 20 calculation results of “1+.” Overall, the significance test results of the GMO algorithm compared with the other 5 algorithms can reach “1+” more than 96% of the time, which further illustrates the advantages of the GMO algorithm.

According to the data tables in Section 3.2, the running times of all algorithms are further counted, as shown in Table 16. The statistical results show that the average running time of the GMO algorithm is similar to the PSO algorithm, and it is lower than that of WOA, BRO, and CSO algorithms. In addition, the benchmark functions corresponding to the minimum, median, and maximum running time of all algorithms are almost the same. It shows that the GMO algorithm has lower time complexity and reliable stability.

### 3.7. Comparative Analysis

In this paper, *D* = 30 is taken as an example, and the experimental results of GMO are compared with the data in the literature [44, 45, 59, 60], as shown in Table 17. It can be seen from the table that the calculation results of the GMO algorithm are significantly better than those of the FSA and KABC algorithms. The performance of the GMO algorithm is similar to that of the FDB-SOS algorithm on unimodal and combinatorial functions, but the GMO algorithm performs better on multimodal functions. Compared with the FDBSFS algorithm, the calculation results of the GMO algorithm are in the same order of magnitude in most functions. This shows that the GMO algorithm is equally competitive with the improved algorithm.

## 4. GMO Algorithm for Engineering Design Problems

In order to verify the applicability of the GMO algorithm on engineering design problems, this section seeks five classical structure design problems, and the GMO algorithm is used to solve the problems. In the experimental process, the design variable is used as the individual's location information in the optimization algorithm, and the calculation model of each problem is used as the objective function. First, the structure design problems are introduced in detail. The problems mainly include three-bar truss design problem, pressure vessel design problem, tension/compression spring design problem, gear train design problem, and cantilever beam design problem. Then, to prove the superiority of the GMO algorithm in solving engineering design problems, the experimental results of the GMO algorithm are compared with the corresponding results of several other algorithms. The results of other algorithms come from literature reports, including KABC [60], DMMFO [61], GOA [62], LSA [63], ALO [38], CS [31], GSA [20], IAPSO [64], CPSO [65], MABGA [66], MBA [67], SOS [68], and CBO [69] algorithms. Finally, all experimental results are analyzed and discussed.

### 4.1. Three-Bar Truss Design Problem

**Figure 11:**
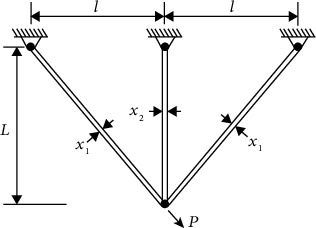
Schematic of three-bar truss mechanism.

Three-bar truss design is a classical optimization problem in mechanics [3, 38], and its mechanism schematic is shown in Figure 11. The problem aims to minimize the volume of a three-bar truss structure, while satisfying the constraints of stress and loading force. The cross-sectional area (*x*_1_,*x*_2_) of the connecting rod is used as the optimization variable, and the optimization objective function is as follows.

(6) $$\begin{array}{c} f_{1}\left(x\right)=\left(2\sqrt{2}x_{1}+x_{2}\right)*l\text{,} \end{array}$$

where *l* is the spacing between the connecting rods, *l* = 100 cm, and *x*_1_,*x*_2_ ∈ [0, 1].

In the process of optimizing variables, the design variables needs to meet the constraints of structural stress, material deflection, and buckling. The three constraint formulas are as follows.

(7) $$\begin{array}{c} \left\{\begin{array}{cc} g_{1}^{1}\left(x\right)=\frac{\sqrt{2}x_{1}+x_{2}}{\sqrt{2}x_{1}^{2}+{2x_{1}x}_{2}}\text{,} & P-\sigma \leq 0\text{,}\\ g_{2}^{1}\left(x\right)=\frac{x_{2}}{\sqrt{2}x_{1}^{2}+{2x_{1}x}_{2}}\text{,} & P-\sigma \leq 0\text{,}\\ g_{3}^{1}\left(x\right)=\frac{1}{\sqrt{2}x_{2}+x_{1}}\text{,} & P-\sigma \leq 0\text{,} \end{array}\right. \end{array}$$

where *P*=2KN/cm^2^, *σ*=2KN/cm^2^.

According to equations (6) and (7), the GMO algorithm is used to solve the three-bar truss problem, and the results are shown in Table 18. Compared with the results of other algorithms, the fitness values of GMO, ALO, and GSA algorithms are optimal, and the solution results satisfy the constraints. It shows that the GMO algorithm is feasible to solve the three-bar truss design problem.

### 4.2. Pressure Vessel Design Problem

**Figure 12:**
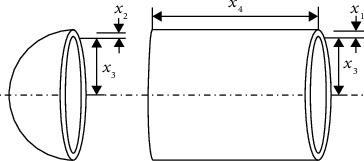
Schematic of pressure vessel structure.

Kannan and Kramer [70] proposed the pressure vessel design problem, which is to minimize the manufacturing cost under the constraints. The structure schematic is shown in Figure 12. This problem consists mainly of 4 design variables, *x*_1_ is the shell thickness of the pressure vessel, *x*_2_ is the thickness of the head, *x*_3_ is the inner ring radius of the pressure vessel, and *x*_4_ is the length of the cylindrical section. The calculation model is as follows.

(8) $$\begin{array}{c} f_{2}\left(x\right)=0.6224x_{1}x_{3}x_{4}+1.7781x_{2}x_{3}+3.1661x_{1}^{2}x_{4}+19.84x_{1}^{2}x_{3}\text{,} \end{array}$$

where *x*_1_, *x*_2_ ∈ [0, 100] and *x*_3_, *x*_4_ ∈ [10, 200], in which *x*_1,_ and *x*_2_ are integer multiples of 0.0625. According to the design specification, the constraint formulas are as follows.

(9) $$\begin{array}{c} \left\{\begin{array}{cc} g_{1}^{2}\left(x\right)={-x}_{1}+0.01930x_{3}, & \leq 0\text{,}\\ g_{2}^{2}\left(x\right)={-x}_{2}+0.00954x_{3}, & \leq 0\text{,}\\ g_{3}^{2}\left(x\right)=-\pi x_{3}^{2}x_{4}-\frac{4}{3}\pi x_{3}^{3}+1960000, & \leq 0\text{,}\\ g_{4}^{2}\left(x\right)=-x_{4}-240, & \leq 0\text{,} \end{array}\right. \end{array}$$

The calculation results of the GMO algorithm and the other 9 algorithms for the pressure vessel design problem are shown in Table 19. The table shows that the results of the KABC, DMMOF, MABGA, and MBA algorithms do not meet the constraints of the variables, which is not desirable. However, the proposed GMO algorithm finds a design with the optimal value identical to LSA, CS, GSA, LAPSO, and CPSO algorithms and satisfies the variable constraints. Therefore, the algorithm is also applicable to solve the pressure vessel design problem.

### 4.3. Tension/Compression Spring Design Problem

**Figure 13:**
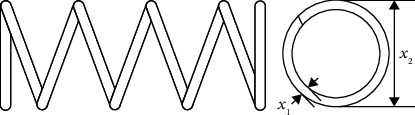
Schematic of the tension/compression spring.

It is an interesting problem to achieve tension/compression spring weight minimization, while satisfying specification and theoretical constraints. This problem was described by Belegundu and Arora [71]. The structure is shown in Figure 13.

The calculate model of tension/compression spring weight is as follows.

(10) $$\begin{array}{c} f_{3}\left(x\right)=\left(x_{3}+2\right)x_{2}x_{1}^{2}\text{,} \end{array}$$

where *x*_1_, *x*_2_, *x*_3_ are the design variables, which are wire diameter, coil diameter, and number of coils, respectively. The value ranges of the design variables are 0.05 ≤ *x*_1_ ≤ 2, 0.25 ≤ *x*_2_ ≤ 1.3, 2 ≤ *x*_3_ ≤ 15, respectively. At the same time, the problem also needs to meet the design theories, such as minimum deflection and shear stress. The specific constraint formulas are as follows.

(11) $$\begin{array}{c} \left\{\begin{array}{cc} g_{1}^{3}\left(x\right)=1-\frac{x_{2}^{3}x_{3}}{71.785x_{1}^{4}},\; & \leq 0\text{,}\\ g_{2}^{3}\left(x\right)=\frac{4x_{2}^{2}-x_{1}x_{2}}{12.566\left(x_{2}x_{1}^{3}-x_{1}^{4}\right)}, & \leq 0\text{,}\\ g_{3}^{3}\left(x\right)=1-\frac{140.45x_{1}}{x_{2}^{2}x_{3}}, & \leq 0\text{,}\\ g_{4}^{3}\left(x\right)=\frac{x_{1}+x_{2}}{1.5}-1, & \leq 0\text{.} \end{array}\right. \end{array}$$

The calculated results of the GMO algorithm for solving the tension/compression spring design problem are shown in Table 20 and compared with the results of 7 other algorithms. It can be seen that the calculated results of all variables meet the requirements of the constraints, and the calculation results of the GMO algorithm are very competitive.

### 4.4. Gear Train Design Problem

**Figure 14:**
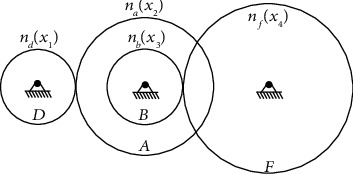
Transmission diagram of the gear train.

The gear train design is a significant engineering design problem in mechanical transmission [72, 73]. The process designs the number of teeth on each gear in the transmission system according to a reasonable transmission ratio. The gear train is shown in Figure 14. The design variables for this problem include the number of teeth of the 4 gears (*x*_1_, *x*_2_, *x*_3_, *x*_4_). The mathematical model is as follows.

(12) $$\begin{array}{c} f_{4}\left(x\right)={\left[\frac{1}{6.931}-\frac{x_{3}x_{2}}{x_{1}x_{4}}\right]}^{2} \end{array}$$

The gear train design problem has a unique solution, and the elements of the solution vector must be integers. The optimization results of the GMO algorithm for the gear train design problem are the same as those of the ALO, IAPSO, and MBA algorithms, as shown in Table 21. It can be seen that the result of the GMO algorithm is optimal and feasible for the problem.

### 4.5. Cantilever Beam Design Problem

**Figure 15:**
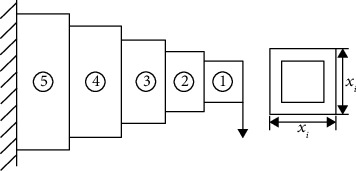
Schematic of the cantilever beam structure.

The cantilever beam design problem is a common engineering problem [74], and its structural diagram is shown in Figure 15. The cantilever beam is mainly composed of 5 sections of square steel with equal wall thickness, and the design variables include the section side length of the 5 sections of square steel (*x*_1_, *x*_2_, *x*_3_, *x*_4_,*x*_5_). The design objective is the minimum weight of the cantilever beam. The calculation model is established in equations (13), and equation (14) is the constraint formula.

(13) $$\begin{array}{c} f_{5}\left(x\right)=0.0624\left(x_{1}+x_{2}+x_{3}+x_{4}+x_{5}\right)\text{,}\\ g^{5}\left(x\right)=\frac{61}{x_{1}^{3}}+\frac{37}{x_{2}^{3}}+\frac{19}{x_{3}^{3}}+\frac{7}{x_{4}^{3}}+\frac{1}{x_{5}^{3}}\text{.} \end{array}$$

The experimental results of the GMO algorithm to optimize the cantilever beam design problem are shown in Table 22. The table shows that the calculation results of all algorithms satisfy the constraints and the optimal fitness values are very close. It is proven that the GMO algorithm obtains satisfactory results.

The comparison results of the above five engineering design problems show that the GMO algorithm has good applicability in practical engineering problems in complex unknown spaces and has achieved satisfactory calculation results. It proves that the GMO algorithm is a promising meta-heuristic optimization algorithm.

## 5. GMO Algorithm for Inverse Kinematics Solution

**Figure 16:**
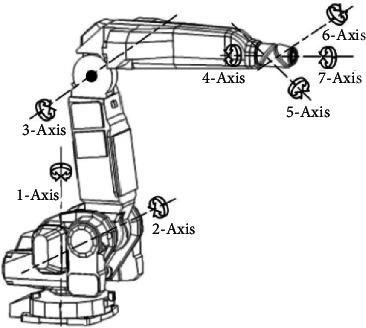
The structure schematic of 7R 6DOF robot.

This paper takes the 7R 6DOF robot as an example to study the inverse kinematics solution of robot by GMO algorithm. The 7R 6DOF robot is composed of 7 rotary joints, which are driven by 6 motors. The robot structure is shown in Figure 16. It has the characteristics of a hollow wrist and flexible movement, which can be used for work in narrow spaces and complex paths. However, the problem of no analytical solution for inverse kinematics limits the field application. Therefore, it may be only feasible to study numerical methods for solving the inverse kinematics of the robot.

### 5.1. Kinematic Modeling of the 7R 6DOF Robot

In this paper, the *D-H* parameter method is used to establish the kinematic model of the 7R 6DOF robot. The forward kinematics model is as follows.

(14) T70=T10∙T21∙T32∙T43∙T54∙T65∙T67,

where _7_^0^ *T* is the pose matrix of the end effector and _*i*_^*i* − 1^ *T* is the coordinate transformation matrix between adjacent links of the robot. The specific transformation matrix is as follows.

(15) Tii−1=cθi−cαisθisαisθiaicθisθicαicθisαicθiaisθi0sαicαi di0001

where *a*_*i*_, *d*_*i*_, *α*_*i*_, and *θ*_*i*_ represent the link length, link offset, link torsion angle, and joint angle, respectively. Among them, *a*_*i*_, *d*_*i*_, *α*_*i*_ are the fixed parameters of the rotary joint robot, and *θ*_*i*_ is the control parameter. This paper takes the IRB5400 robot with a 7R 6DOF structure as an example, and its *D*-*H* parameters are shown in Table 23 [75].

According to the input robot joint angles, the pose matrix of the robot end position is solved through the forward kinematics formula and *D*-*H* parameters. The pose matrix of the robot end position is as follows.

(16) T70=nxoxnyoyαxpxαypynzoz00αzpz01=noαp0001,

where *n*_*x*_, *n*_*y*_, *n*_*z*_, *o*_*x*_,*o*_*y*_, *o*_*z*_, *α*_*x*_, *α*_*y*_, *α*_*z*_ represent the rotational elements of the pose matrix and *p*_*x*_, *p*_*y*_, *p*_*z*_ represent the elements of position vector.

In order to realize the step-by-step optimization of the GMO algorithm in the inverse kinematics solution process, the objective function is designed in equation (17), which is the difference between the expected value and the actual value of the pose matrix.

(17) $$\begin{array}{c} f\left(x\right)=sum\left(\left|n-n^{\wedge }\right|+\left|o-o^{\wedge }\right|+\left|\alpha -\alpha^{\wedge }\right|+\gamma \left|p-p^{\wedge }\right|\right) \end{array}$$

where *n* ^∧^, *o* ^∧^, *α* ^∧^, *p* ^∧^ represent the rotational and position vectors of the expected pose matrix and *γ* is the adjustment factor.

### 5.2. Experiment and Result Analysis

According to the forward kinematics model and objective function of the 7R 6DOF robot, the inverse kinematics experiment of the GMO algorithm takes the joint angle of the robot as the optimization variable and the desired end pose as the optimization goal. Then, to prove the GMO algorithm's computational performance in solving the inverse kinematics of the robot, the experimental results of the GMO algorithm are compared with the WOA, PSO, BRO, CSO, and ABC algorithms. In the experiment, two pose matrices of the robot end position are randomly selected as the test points, and the pose matrix is shown in Table 24. The population size *N* = 100, the maximum number *T* = 500, and the adjustment factor *γ* = 1.

**Figure 17:**
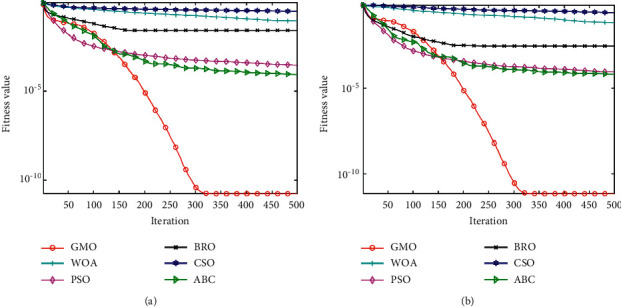
Average convergence curve of 6 algorithms. (a) Test point 1. (b) Test point 2.

During the experiment, in order to avoid the influence of accidental results, 50 independent experiments are conducted at each test point, and the best, worst, mean, and standard deviation of each algorithm's convergence results are recorded. The results are shown in Table 25. It can be seen from the table that the average value of the GMO algorithm has reached 1.0*E* − 11 on two test points, which is at least 5 orders of magnitude better than other algorithms. The best, worst, and standard deviation values are also better than those of the other 5 algorithms. The average convergence curve is shown in Figure 17. Its shows that the GMO algorithm has fast convergence speed and high accuracy.

However, the effectiveness of solving the inverse kinematics problem can be more directly verified by the independent errors of each element in the pose matrix. As shown in Table 26, the independent errors of each element in the pose matrix are calculated. It can be seen that the error of each element in the solution result by the GMO algorithm is less than 1.0*E* − 15, which is higher than the minimum error in other algorithms. The experimental results verify the feasibility of the GMO algorithm to solve the inverse kinematics problem.

In recent years, scholars have made a lot of valuable explorations to solve the inverse kinematics of robots through intelligent methods. This paper counted the experimental results in the literature and compared them with the solution results of the GMO algorithm, as shown in Table 27. It can be seen that scholars have achieved more research results on the problem of solving the robot end position. However, there are fewer studies on more complex pose problems, and the results are less accurate. The GMO algorithm is applied to solve the inverse kinematic pose problem of a complex 7R 6DOF robot. The average solution result of 50 experiments is 1.68*E* − 11, which shows that the GMO algorithm has a high solution accuracy and excellent applicability.

## 6. Conclusion

In this paper, the wild geese migration optimization (GMO) algorithm is inspired by the behavior of wild geese migration. The mathematical optimization model of GMO algorithm is designed by simulating the special migration process of the wild geese, which has the advantages of simple structure and few parameters. In order to verify the optimization ability of the GMO algorithm, the 29 stable benchmark functions from CEC2017 are used for 50 experiments, respectively. The primary performance evaluation indicators are the mean, standard deviation, significance test results, and the algorithm's running time. The test results of the GMO algorithm and WOA, PSO, BRO, CSO, and ABC algorithms are statistically analyzed. It can be seen that the GMO algorithm has apparent advantages in computing performance and can better seek a balance between exploitation and exploration. It is a sufficiently competitive optimization algorithm.

In addition, the GMO algorithm is used to solve five engineering optimization problems, and the solution results are compared with the results provided in other studies. The comparison results show that the GMO algorithm obtains excellent solution results, and the experimental results meet the constraints of engineering optimization problems. This shows that the GMO algorithm has satisfactory computing performance and universality in the face of unknown space and complex practical problems. Finally, the GMO algorithm is applied to the inverse kinematic pose problem of the 7R 6DOF robot. The experimental results show that the average solution accuracy of the end pose of the GMO algorithm reaches 1.0*E* − 11, which is at least 5 orders of magnitude higher than that of the comparison algorithm. The GMO algorithm provides a new solution for the inverse kinematics of the complex 7R 6DOF robot, showing that the algorithm has strong practicability and good development prospects.

In future work, we will study the independent optimization mechanism of the migration groups in the GMO algorithm and the multiobjective optimization problem of the GMO algorithm and explore more valuable practical application cases.

## Figures and Tables

**Algorithm 1:**
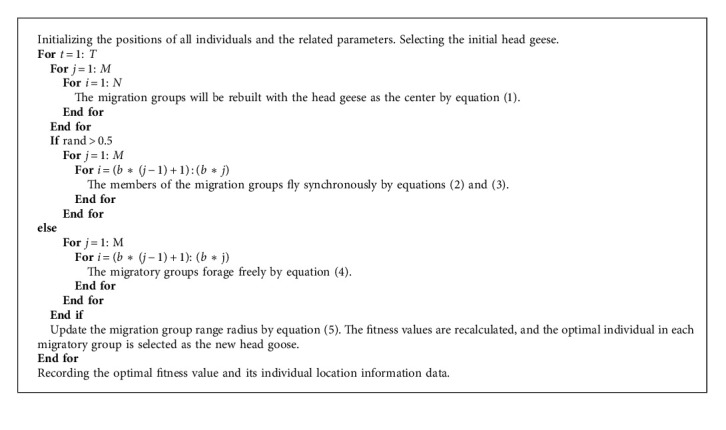
The pseudocode of GMO.

**Table 1:** Benchmark functions.

Function type	Function name		Range	*Fi ∗*
Unimodal functions	*F*1	Shifted and rotated bent cigar function	[−100, 100]	100
	*F*3	Shifted and rotated Zakharov function	[−100, 100]	300

Multimodal functions	*F*4	Shifted and rotated Rosenbrock's function	[−100, 100]	400
	*F*5	Shifted and rotated Rastrigin's function	[−100, 100]	500
	*F*6	Shifted and rotated expanded Schaffer's *F*6 function	[−100, 100]	600
	*F*7	Shifted and rotated Lunacek Bi_Rastrigin function	[−100, 100]	700
	*F*8	Shifted and rotated noncontinuous Rastrigin's function	[−100, 100]	800
	*F*9	Shifted and rotated Lévy function	[−100, 100]	900
	*F*10	Shifted and rotated Schwefel's function	[−100, 100]	1000

Hybrid functions	*F*11	Hybrid function 1 (*N* = 3)	[−100, 100]	1100
	*F*12	Hybrid function 2 (*N* = 3)	[−100, 100]	1200
	*F*13	Hybrid function 3 (*N* = 3)	[−100, 100]	1300
	*F*14	Hybrid function 4 (*N* = 4)	[−100, 100]	1400
	*F*15	Hybrid function 5 (*N* = 4)	[−100, 100]	1500
	*F*16	Hybrid function 6 (*N* = 4)	[−100, 100]	1600
	*F*17	Hybrid function 6 (*N* = 5)	[−100, 100]	1700
	*F*18	Hybrid function 6 (*N* = 5)	[−100, 100]	1800
	*F*19	Hybrid function 6 (*N* = 5)	[−100, 100]	1900
	*F*20	Hybrid function 6 (*N* = 6)	[−100, 100]	2000

Composition functions	*F*21	Composition function 1 (*N* = 3)	[−100, 100]	2100
	*F*22	Composition function 2 (*N* = 3)	[−100, 100]	2200
	*F*23	Composition function 3 (*N* = 4)	[−100, 100]	2300
	*F*24	Composition function 4 (*N* = 4)	[−100, 100]	2400
	*F*25	Composition function 5 (*N* = 5)	[−100, 100]	2500
	*F*26	Composition function 6 (*N* = 5)	[−100, 100]	2600
	*F*27	Composition function 7 (*N* = 6)	[−100, 100]	2700
	*F*28	Composition function 8 (*N* = 6)	[−100, 100]	2800
	*F*29	Composition function 9 (*N* = 3)	[−100, 100]	2900
	*F*30	Composition function 10 (*N* = 3)	[−100, 100]	3000

**Table 2:** Parameters of all algorithms.

Algorithms	Parameters
GMO	*M* = 20, *L* = (*ud* − *ld*)/*N*
WOA	*a* is linearly decreased from 2 to 0, *b*=1
PSO	The inertia is 0.8, the two learning factors are 0.5
BRO	The damage threshold is 3
CSO	*N*_*r*_=0.2*N*, *N*_*h*_=0.6*N*, *N*_*c*_=*N* − *N*_*r*_ − *N*_*h*_, *N*_*m*_=0.1*N*, *G*=10, *FL* ∈ [0.4,1]
ABC	The number of food sources is 50, the limit is 20

**Table 3:** The results of the algorithms on unimodal and multimodal functions in *D* = 10.

Function			GMO	WOA	PSO	BRO	CSO	ABC
*F*1	Mean		1778.537	1070535	8.64*E* + 08	1.56*E* + 09	1.22*E* + 10	595859.2
	Std.		1847.493	1327311	6.69*E* + 08	3.77*E* + 08	2.76*E* + 09	447757.2
	Time		0.183993	0.189141	0.150858	2.233721	0.275354	0.089319
	Rank		1	3	4	5	6	2
	Test	*p*	*—*	7.07*E *−* *18	7.07*E *−* *18	7.07*E *−* *18	7.07*E *−* *18	7.07*E *−* *18
		*h*	*—*	1	1	1	1	1

*F*3	Mean		5.01*E *−* *13	402.1686	8723.172	1314.335	12339.67	14.95538
	Std.		2.11*E *−* *12	258.4504	5231.26	404.7079	2742.961	10.33418
	Time		0.182382	0.189662	0.149261	2.244138	0.278865	0.089135
	Rank		1	3	5	4	6	2
	Test	*p*	*—*	2.95*E *−* *18	2.95*E *−* *18	2.95*E *−* *18	2.95*E *−* *18	2.95*E *−* *18
		*h*	*—*	1	1	1	1	1

*F*4	Mean		2.714653	74.34308	86.33005	120.5624	796.107	11.22363
	Std.		1.691115	1.738098	56.63311	27.75917	309.0472	17.91742
	Time		0.183079	0.190755	0.148013	2.260358	0.27832	0.090793
	Rank		1	3	4	5	6	2
	Test	*p*	*—*	1.21*E *−* *131	7.07*E *−* *18	2.49*E *−* *33	2.13*E *−* *23	1.6*E *−* *11
		*h*	*—*	1	1	1	1	1

*F*5	Mean		19.64816	60.35007	48.12616	56.69372	108.7148	30.34673
	Std.		8.114833	20.74515	7.503261	13.57173	12.00771	6.384102
	Time		0.202584	0.20961	0.169415	2.268328	0.285728	0.100563
	Rank		1	5	3	4	6	2
	Test	*p*	—	1.88*E *−* *19	3.10*E *−* *33	1.37*E *−* *27	2.55*E *−* *60	6.72*E *−* *11
		*h*	—	1	1	1	1	1

*F*6	Mean		1.090706	37.46023	29.75035	32.48941	45.93866	5.483345
	Std.		1.452416	11.4719	14.76915	7.580359	5.717901	2.554292
	Time		0.25974	0.266008	0.223852	2.303247	0.352943	0.129438
	Rank		1	5	3	4	6	2
	Test	*p*	—	7.07*E *−* *18	7.07*E *−* *18	7.07*E *−* *18	7.07*E *−* *18	2.38*E *−* *14
		*h*	—	1	1	1	1	1

*F*7	Mean		31.09322	87.20033	97.05758	61.33053	144.8	47.98111
	Std.		8.94308	26.17437	14.06498	17.80283	12.16385	5.388259
	Time		0.211016	0.214148	0.177618	2.269094	0.293629	0.103522
	Rank		1	4	5	3	6	2
	Test	*P*	—	4.27*E *−* *21	4.75*E *−* *44	1.31*E *−* *16	7.77*E *−* *70	1.53*E *−* *18
		*H*	—	1	1	1	1	1

*F*8	Mean		21.27218	69.84663	55.49274	34.09814	57.225	23.67172
	Std.		6.95421	13.90796	8.125503	11.40441	5.135724	5.611597
	Time		0.203695	0.208884	0.170855	2.321535	0.301693	0.099989
	Rank		1	6	4	3	5	2
	Test	*p*	—	8.97*E *−* *17	1.08*E *−* *40	1.71*E *−* *09	5.35*E *−* *48	0.0605
		*h*	—	1	1	1	1	0

*F*9	Mean		1.319961	698.5982	239.4674	248.3575	976.1072	2.508042
	Std.		2.956793	387.5305	140.1229	165.4117	234.7014	2.497579
	Time		0.216275	0.217557	0.179534	2.251371	0.294001	0.105641
	Rank		1	5	3	4	6	2
	Test	*p*	—	7.8*E *−* *18	6.92*E *−* *18	6.92*E *−* *18	6.92*E *−* *18	2.54*E *−* *07
		*h*	—	1	1	1	1	1

*F*10	Mean		678.6022	1015.777	1101.43	1236.56	2209.537	1401.925
	Std.		256.9167	407.7181	234.8599	256.3246	266.9975	148.902
	Time		0.220632	0.226075	0.184279	2.267796	0.304015	0.111241
	Rank		1	2	3	4	6	5
	Test	*p*	—	3.90*E *−* *06	1.38*E *−* *13	1.57*E *−* *18	7.5*E *−* *18	3.32E-17
		*h*	—	1	1	1	1	1

Average rank			1	3.88	3.88	4	5.88	2.38
Overall rank			1	3	3	5	6	2

**Table 4 tab4:** The results of the algorithms on hybrid functions in *D* = 10.

Function			GMO	WOA	PSO	BRO	CSO	ABC
*F*11	Mean		3.42*E* + 01	9.19*E* + 01	2.86*E* + 02	6.46*E* + 01	2.65*E* + 03	3.62*E* + 01
	Std.		2.56*E* + 01	7.95*E* + 01	1.21*E* + 02	2.95*E* + 01	1.34*E* + 03	2.19*E* + 01
	Time		1.98*E *−* *01	2.04*E *−* *01	1.62*E *−* *01	2.29*E* + 00	2.89*E *−* *01	9.63*E *−* *02
	Rank		1	4	5	3	6	2
	Test	*p*	—	2.14*E *−* *06	1.14*E *−* *17	3.42*E *−* *08	7.07*E *−* *18	0.371991
		*h*	—	1	1	1	1	0

*F*12	Mean		4.58*E* + 03	2.18*E* + 05	7.36*E* + 06	2.97*E* + 05	8.37*E* + 07	2.69*E* + 05
	Std.		4.23*E* + 03	1.32*E* + 05	5.20*E* + 06	1.70*E* + 05	6.02*E *−* *08	2.62*E* + 05
	Time		1.98*E *−* *01	2.05*E *−* *01	1.66*E *−* *01	2.24*E* + 00	2.86*E *−* *01	9.87*E *−* *02
	Rank		1	2	5	4	6	3
	Test	*p*	—	1.84*E *−* *17	7.07*E *−* *18	7.07*E *−* *18	3.31*E *−* *20	8.99*E *−* *17
		*h*	—	1	1	1	1	1

*F*13	Mean		9.08*E* + 02	1.08*E* + 04	7.46*E* + 04	1.00*E* + 04	1.69*E* + 07	8.89*E* + 03
	Std.		5.52*E* + 02	3.63*E* + 03	6.05*E* + 04	1.27*E* + 03	9.81*E* + 06	3.48*E* + 03
	Time		2.03*E *−* *01	2.08*E *−* *01	1.68*E *−* *01	2.24*E* + 00	2.94*E *−* *01	1.01*E *−* *01
	Rank		1	4	5	3	6	2
	Test	*p*	—	7.07*E *−* *18	7.07*E *−* *18	7.97*E *−* *18	6.23*E *−* *18	1.27*E *−* *21
		*h*	—	1	1	1	1	1

*F*14	Mean		5.86*E* + 01	1.53*E* + 02	4.70*E* + 02	1.09*E* + 02	6.92*E* + 03	5.95*E* + 01
	Std.		1.32*E* + 01	1.32*E* + 02	6.31*E* + 02	8.12*E* + 01	1.41*E* + 03	1.14*E* + 01
	Time		2.14*E *−* *01	2.20*E *−* *01	1.78*E *−* *01	2.26*E* + 00	2.90*E *−* *01	1.07*E *−* *01
	Rank		1	4	5	3	6	2
	Test	*p*	—	9.46*E *−* *10	7.97*E *−* *18	1.03*E *−* *08	3.5*E *−* *19	0.7158
		*h*	—	1	1	1	1	0

*F*15	Mean		1.73*E* + 02	4.54*E* + 03	6.38*E* + 03	4.36*E* + 03	4.04*E* + 04	2.54*E* + 02
	Std.		6.84*E* + 01	2.63*E* + 03	3.26*E* + 03	2.60*E* + 03	2.60*E* + 04	1.90*E* + 02
	Time		1.91*E *−* *01	1.95*E *−* *01	1.57*E *−* *01	2.24*E* + 00	2.87*E *−* *01	9.21*E *−* *02
	Rank		1	4	5	3	6	2
	Test	*p*	—	7.5*E *−* *18	4.19*E *−* *18	7.07*E *−* *18	6.38*E *−* *18	0.055744
		*h*	—	1	1	1	1	0

*F*16	Mean		9.29*E* + 01	3.07*E* + 02	1.41*E* + 02	2.38*E* + 02	6.86*E* + 02	1.20*E* + 02
	Std.		7.72*E* + 01	1.20*E* + 02	7.74*E* + 01	1.03*E* + 02	1.16*E* + 02	5.80*E* + 01
	Time		2.06*E *−* *01	2.10*E *−* *01	1.70*E *−* *01	2.27*E* + 00	2.96*E *−* *01	9.97*E *−* *02
	Rank		1	5	3	4	6	2
	Test	*p*	—	9.42*E *−* *14	0.000925	3.02*E *−* *10	7.07*E *−* *18	0.008367
		*h*	—	1	1	1	1	1

*F*17	Mean		5.24*E* + 01	4.84*E* + 01	9.59*E* + 01	5.38*E* + 01	2.27*E* + 02	6.57*E* + 01
	Std.		1.42*E* + 01	1.31*E* + 01	1.78*E* + 01	1.32*E* + 01	4.60*E* + 01	7.36*E* + 00
	Time		2.56*E *−* *01	2.59*E *−* *01	2.24*E *−* *01	2.37*E* + 00	3.38*E *−* *01	1.27*E *−* *01
	Rank		2	1	5	3	6	4
	Test	*p*	—	0.1386	4.30*E *−* *24	0.6165	1.92*E *−* *33	1.11*E *−* *07
		*h*	—	0	1	0	1	1

*F*18	Mean		1.75*E* + 02	1.57*E* + 03	9.77*E* + 04	1.35*E* + 03	2.67*E* + 07	4.63*E* + 03
	Std.		1.11*E* + 02	4.87*E* + 03	8.29*E* + 04	2.27*E* + 03	1.17*E* + 07	4.39*E* + 03
	Time		2.04*E *−* *01	2.08*E *−* *01	1.69*E *−* *01	2.27*E* + 00	2.94*E *−* *01	9.96*E *−* *02
	Rank		1	3	5	2	6	4
	Test	*p*	—	5.98*E *−* *17	7.07*E *−* *18	1.84*E *−* *17	2.76*E *−* *18	3.32*E *−* *17
		*h*	—	1	1	1	1	1

*F*19	Mean		7.40*E* + 01	3.27*E* + 04	8.06*E* + 03	5.59*E* + 03	1.21*E* + 06	8.46*E* + 01
	Std.		4.43*E* + 01	2.87*E* + 04	1.70*E* + 04	2.72*E* + 03	1.29*E* + 06	8.30*E* + 01
	Time		5.31*E *−* *01	5.33*E *−* *01	4.94*E *−* *01	2.60*E* + 00	5.83*E *−* *01	2.67*E *−* *01
	Rank		1	5	4	3	6	2
	Test	*p*	—	7.07*E *−* *18	7.07*E *−* *18	3.36*E *−* *19	7.06*E *−* *18	0.612368
		*h*	—	1	1	1	1	0

*F*20	Mean		5.89*E* + 01	1.06*E* + 02	1.01*E* + 02	1.05*E* + 02	3.42*E* + 02	1.05*E* + 02
	Std.		2.29*E* + 01	2.82*E* + 01	1.76*E* + 01	4.32*E* + 01	5.85*E* + 01	2.59*E* + 01
	Time		2.57*E *−* *01	2.62*E *−* *01	2.25*E *−* *01	2.32*E* + 00	3.41*E *−* *01	1.29*E *−* *01
	Rank		1	5	2	4	6	3
	Test	*p*	—	3.43*E *−* *13	2.52*E *−* *13	9.06*E *−* *10	7.22*E *−* *41	1.99*E *−* *15
		*h*	—	1	1	1	1	1

Average rank			1.1	3.7	4.4	3.2	6	2.6
Overall rank			1	4	5	3	6	2

**Table 5 tab5:** The results of the algorithms on composition functions in *D* = 10.

Function			GMO	WOA	PSO	BRO	CSO	ABC
*F*21	Mean		1.98*E* + 02	2.19*E* + 02	2.47*E* + 02	1.19*E* + 02	2.77*E* + 02	1.19*E* + 02
	Std.		4.61*E* + 01	6.15*E* + 01	2.91*E* + 01	9.65*E* + 00	3.93*E* + 01	1.98*E* + 01
	Time		2.65*E *−* *01	2.68*E *−* *01	2.30*E *−* *01	2.33*E* + 00	3.50*E *−* *01	1.31*E *−* *01
	Rank		3	4	5	2	6	1
	Test	*p*	—	6.22*E *−* *06	1.55*E *−* *15	3.56*E *−* *08	4.9*E *−* *13	2.4*E *−* *08
		*h*	—	1	1	1	1	1

*F*22	Mean		1.03*E* + 02	1.17*E* + 02	1.56*E* + 02	1.82*E* + 02	1.01*E* + 03	1.09*E* + 02
	Std.		2.11*E* + 00	8.03*E* + 00	1.80*E* + 01	3.34*E* + 01	1.93*E* + 02	2.66*E* + 00
	Time		2.99*E *−* *01	3.01*E *−* *01	2.63*E *−* *01	2.38*E* + 00	3.59*E *−* *01	1.48*E *−* *01
	Rank		1	3	4	5	6	2
	Test	*p*	—	3.13*E *−* *17	7.07*E *−* *18	3.46*E *−* *14	7.07*E *−* *18	3.98*E *−* *15
		*h*	—	1	1	1	1	1

*F*23	Mean		3.22*E* + 02	3.47*E* + 02	3.92*E* + 02	3.84*E* + 02	4.89*E* + 02	3.24*E* + 02
	Std.		7.40*E* + 00	2.22*E* + 01	3.02*E* + 01	2.09*E* + 01	2.80*E* + 01	7.61*E* + 00
	Time		3.12*E *−* *01	3.15*E *−* *01	2.72*E *−* *01	2.39*E* + 00	3.68*E *−* *01	1.55*E *−* *01
	Rank		1	3	5	4	6	2
	Test	*p*	—	5.4*E *−* *11	1.37*E *−* *17	8.46*E *−* *18	7.07*E *−* *18	0.210851
		*h*	—	1	1	1	1	0

*F*24	Mean		3.24*E* + 02	3.74*E* + 02	3.82*E* + 02	2.98*E* + 02	4.88*E* + 02	2.76*E* + 02
	Std.		7.56*E* + 01	1.97*E* + 01	1.04*E* + 01	1.31*E* + 02	4.39*E* + 01	9.11*E* + 01
	Time		3.24*E *−* *01	3.29*E *−* *01	2.86*E *−* *01	2.43*E* + 00	3.87*E *−* *01	1.62*E *−* *01
	Rank		3	4	5	2	6	1
	Test	*p*	—	1.1*E *−* *13	1.84*E *−* *17	0.037035	7.89*E *−* *16	1.08*E *−* *06
		*h*	—	1	1	1	1	1

*F*25	Mean		4.22*E* + 02	4.24*E* + 02	4.70*E* + 02	4.72*E* + 02	8.81*E* + 02	4.33*E* + 02
	Std.		2.36*E* + 01	1.42*E* + 01	3.03*E* + 01	1.66*E* + 01	9.08*E* + 01	2.02*E* + 01
	Time		2.89*E *−* *01	2.95*E *−* *01	2.53*E *−* *01	2.36*E* + 00	3.57*E *−* *01	1.44*E *−* *01
	Rank		1	2	4	5	6	3
	Test	*p*	—	0.446195	1.41*E *−* *10	4.96*E *−* *15	7.06*E *−* *18	0.000255
		*h*	—	0	1	1	1	1

*F*26	Mean		3.11*E* + 02	4.27*E* + 02	4.48*E* + 02	6.23*E* + 02	1.49*E* + 03	3.29*E* + 02
	Std.		2.36*E* + 01	5.36*E* + 01	2.20*E* + 01	5.72*E* + 01	1.74*E* + 02	4.93*E* + 01
	Time		3.43*E *−* *01	3.48*E *−* *01	3.09*E *−* *01	2.42*E* + 00	3.97*E *−* *01	1.72*E *−* *01
	Rank		1	3	4	5	6	2
	Test	*p*	—	3.3*E *−* *17	8.91*E *−* *18	7*E *−* *18	6.55*E *−* *19	2.2*E *−* *07
		*h*	—	1	1	1	1	1

*F*27	Mean		3.93*E* + 02	4.11*E* + 02	4.51*E* + 02	4.64*E* + 02	5.15*E* + 02	3.96*E* + 02
	Std.		2.78*E* + 00	1.53*E* + 01	2.72*E* + 01	2.03*E* + 01	4.80*E* + 01	3.52*E* + 00
	Time		3.55*E *−* *01	3.60*E *−* *01	3.16*E *−* *01	2.42*E* + 00	4.23*E *−* *01	1.78*E *−* *01
	Rank		1	3	4	5	6	2
	Test	*p*	—	1.43*E *−* *16	1.21*E *−* *17	7.06*E *−* *18	7.02*E *−* *18	1.43*E *−* *05
		*h*	—	1	1	1	1	1

*F*28	Mean		3.23*E* + 02	4.14*E* + 02	4.44*E* + 02	4.78*E* + 02	6.51*E* + 02	5.05*E* + 02
	Std.		4.20*E* + 01	1.01*E* + 01	5.94*E* + 00	5.40*E* + 01	7.16*E* + 00	1.26*E* + 02
	Time		3.30*E *−* *01	3.34*E *−* *01	2.92*E *−* *01	2.43*E* + 00	3.91*E *−* *01	1.64*E *−* *01
	Rank		1	2	3	4	6	5
	Test	*p*	—	3.13*E *−* *17	7.06*E *−* *18	8.48*E *−* *17	4.73*E *−* *20	1.76*E *−* *13
		*h*	—	1	1	1	1	1

*F*29	Mean		2.63*E* + 02	3.11*E* + 02	2.89*E* + 02	3.41*E* + 02	6.28*E* + 02	3.11*E* + 02
	Std.		2.39*E* + 01	3.23*E* + 01	3.05*E* + 01	3.35*E* + 01	9.28*E* + 01	1.84*E* + 01
	Time		3.19*E *−* *01	3.26*E *−* *01	2.80*E *−* *01	2.39*E* + 00	3.95*E *−* *01	1.59*E *−* *01
	Rank		1	3	2	5	6	4
	Test	*p*	—	5.92*E *−* *11	1.11*E *−* *06	3.38*E *−* *16	7.07*E *−* *18	1.16*E *−* *13
		*h*	—	1	1	1	1	1

*F*30	Mean		5.15*E* + 04	3.83*E* + 06	3.32*E* + 06	1.49*E* + 06	1.55*E* + 07	4.38*E* + 05
	Std.		2.43*E* + 05	2.31*E* + 06	2.95*E* + 06	1.30*E* + 06	9.22*E* + 06	6.85*E* + 05
	Time		5.93*E *−* *01	5.95*E *−* *01	5.52*E *−* *01	2.66*E* + 00	6.43*E *−* *01	2.99*E *−* *01
	Rank		1	4	3	5	6	2
	Test	*p*	—	2.95*E *−* *17	6.26*E *−* *17	7.55*E *−* *17	6.93*E *−* *18	1.16*E *−* *13
		*h*	—	1	1	1	1	1

Average rank			1.4	3.2	3.9	4.2	6	2.4
Overall rank			1	3	4	5	6	2

**Table 6 tab6:** The results of the algorithms on unimodal and multimodal functions in *D* = 30.

Function			GMO	WOA	PSO	BRO	CSO	ABC
*F*1	Mean		2.96*E* + 04	4.54*E* + 08	1.18*E* + 10	2.16*E* + 10	5.92*E* + 10	4.40*E* + 08
	Std.		2.13*E* + 04	2.39*E* + 08	3.13*E* + 09	1.98*E* + 09	4.55*E* + 09	1.95*E* + 08
	Time		4.36*E *−* *01	4.61*E *−* *01	3.52*E *−* *01	3.70*E* + 00	6.66*E *−* *01	2.51*E *−* *01
	Rank		1	3	4	5	6	2
	Test	*p*	—	7.07*E *−* *18	7.07*E *−* *18	7.07*E *−* *18	7.07*E *−* *18	7.07*E *−* *18
		*h*	—	1	1	1	1	1

*F*3	Mean		2.52*E* + 04	2.48*E* + 05	2.06*E* + 05	4.87*E* + 04	8.79*E* + 04	1.73*E* + 04
	Std.		9.19*E* + 03	2.82*E* + 04	4.61*E* + 04	5.23*E* + 03	5.55*E* + 03	4.38*E* + 03
	Time		4.34*E *−* *01	4.59*E *−* *01	3.53*E *−* *01	3.64*E* + 00	6.72*E *−* *01	2.43*E *−* *01
	Rank		2	6	5	3	4	1
	Test	*p*	—	7.07*E *−* *18	1.04*E *−* *32	6.43*E *−* *26	7.07*E *−* *18	5.78*E *−* *07
		*h*	—	1	1	1	1	1

*F*4	Mean		1.17*E* + 02	2.94*E* + 02	1.77*E* + 03	5.40*E* + 03	1.62*E* + 04	2.76*E* + 02
	Std.		1.96*E* + 01	8.22*E* + 01	1.12*E* + 03	6.57*E* + 02	2.75*E* + 03	5.96*E* + 01
	Time		4.34*E *−* *01	4.52*E *−* *01	3.51*E *−* *01	3.67*E* + 00	6.58*E *−* *01	2.46*E *−* *01
	Rank		1	3	4	5	6	2
	Test	*p*	—	9.55*E *−* *21	7.07*E *−* *18	1.92*E *−* *46	8.77*E *−* *40	1.26*E *−* *25
		*h*	—	1	1	1	1	1

*F*5	Mean		1.21*E* + 02	3.15*E* + 02	3.36*E* + 02	3.19*E* + 02	4.76*E* + 02	1.85*E* + 02
	Std.		3.37*E* + 01	5.59*E* + 01	3.36*E* + 01	3.37*E* + 01	2.67*E* + 01	2.81*E* + 01
	Time		4.94*E *−* *01	5.23*E *−* *01	4.11*E *−* *01	3.62*E* + 00	7.01*E *−* *01	3.41*E *−* *01
	Rank		1	3	5	4	6	2
	Test	*p*	—	7.07*E *−* *18	1.19*E *−* *53	2.09*E *−* *50	6.45*E *−* *78	2.61*E *−* *17
		*h*	—	1	1	1	1	1

*F*6	Mean		3.07*E* + 01	7.26*E* + 01	5.52*E* + 01	7.09*E* + 01	9.22*E* + 01	2.73*E* + 01
	Std.		9.70*E* + 00	7.50*E* + 00	9.96*E* + 00	7.91*E* + 00	3.56*E* + 00	7.19*E* + 00
	Time		7.21*E *−* *01	7.43*E *−* *01	6.35*E *−* *01	3.92*E* + 00	9.56*E *−* *01	5.00*E *−* *01
	Rank		2	5	3	4	6	1
	Test	*p*	—	4.39*E *−* *43	7.03*E *−* *22	9.16*E *−* *41	2.79*E *−* *47	0.0497
		*h*	—	1	1	1	1	0

*F*7	Mean		1.79*E* + 02	5.20*E* + 02	6.40*E* + 02	4.71*E* + 02	7.95*E* + 02	3.08*E* + 02
	Std.		3.81*E* + 01	8.95*E* + 01	7.86*E* + 01	6.71*E* + 01	2.62*E* + 01	2.67*E* + 01
	Time		5.17*E *−* *01	5.40*E *−* *01	4.33*E *−* *01	3.71*E* + 00	7.30*E *−* *01	3.23*E *−* *01
	Rank		1	4	5	3	6	2
	Test	*p*	—	7.07*E *−* *18	7.07*E *−* *18	7.07*E *−* *18	7.07*E *−* *18	1.37*E *−* *17
		*h*	—	1	1	1	1	1

*F*8	Mean		1.22*E* + 02	2.11*E* + 02	3.15*E* + 02	2.45*E* + 02	3.86*E* + 02	1.48*E* + 02
	Std.		3.09*E* + 01	4.43*E* + 01	2.88*E* + 01	3.48*E* + 01	1.69*E* + 01	2.84*E* + 01
	Time		5.10*E *−* *01	5.35*E *−* *01	4.26*E *−* *01	3.73*E* + 00	7.11*E *−* *01	3.53*E *−* *01
	Rank		1	3	5	4	6	2
	Test	*p*	—	1.18*E *−* *19	3.56*E *−* *54	4.04*E *−* *34	7.40*E *−* *62	1.96*E *−* *05
		*h*	—	1	1	1	1	1

*F*9	Mean		1.36*E* + 03	6.11*E* + 03	7.70*E* + 03	6.68*E* + 03	1.40*E* + 04	1.29*E* + 03
	Std.		7.41*E* + 02	1.43*E* + 03	2.07*E* + 03	1.37*E* + 03	1.21*E* + 03	6.02*E* + 02
	Time		5.14*E *−* *01	5.38*E *−* *01	4.32*E *−* *01	3.74*E* + 00	7.25*E *−* *01	3.10*E *−* *01
	Rank		2	3	5	4	6	1
	Test	*p*	—	7.5*E *−* *18	7.07*E *−* *18	7.07*E *−* *18	7.07*E *−* *18	0.790692
		*h*	—	1	1	1	1	0

*F*10	Mean		4.56*E* + 03	6.04*E* + 03	7.30*E* + 03	7.32*E* + 03	8.45*E* + 03	7.39*E* + 03
	Std.		8.56*E* + 02	6.83*E* + 02	5.19*E* + 02	3.93*E* + 02	3.15*E* + 02	3.26*E* + 02
	Time		5.61*E *−* *01	5.81*E *−* *01	4.82*E *−* *01	3.79*E* + 00	7.43*E *−* *01	5.03*E *−* *01
	Rank		1	2	3	4	6	5
	Test	*p*	—	1.09*E *−* *15	1.84*E *−* *17	1.21*E *−* *17	1.15*E *−* *43	4.36*E *−* *31
		*h*	—	1	1	1	1	1

Average rank			1.11	3	4.67	4	5.78	2.44
Overall rank			1	3	5	4	6	2

**Table 7 tab7:** The results of the algorithms on hybrid functions in *D* = 30.

Function			GMO	WOA	PSO	BRO	CSO	ABC
*F*11	Mean		2.14*E* + 02	1.90*E* + 03	4.90*E* + 03	1.20*E* + 03	8.46*E* + 03	3.28*E* + 02
	Std.		6.42*E* + 01	4.00*E* + 02	1.97*E* + 03	1.86*E* + 02	1.18*E* + 03	7.92*E* + 01
	Time		4.75*E *−* *01	5.01*E *−* *01	3.92*E *−* *01	3.75*E* + 00	7.02*E *−* *01	2.68*E *−* *01
	Rank		1	4	5	3	6	2
	Test	*p*	—	7.03*E *−* *34	5.34*E *−* *22	3.50*E *−* *42	1.15*E *−* *43	5.92*E *−* *11
		*h*	*—*	1	1	1	1	1

*F*12	Mean		9.13*E* + 06	2.04*E* + 08	1.19*E* + 09	4.26*E* + 09	1.69*E* + 10	1.23*E* + 08
	Std.		7.86*E* + 06	1.06*E* + 08	7.39*E* + 08	6.93*E* + 08	2.06*E* + 09	8.41*E* + 07
	Time		3.27*E *−* *01	3.35*E *−* *01	2.96*E *−* *01	2.62*E* + 00	4.00*E *−* *01	1.59*E *−* *01
	Rank		1	3	4	5	6	2
	Test	*p*	*—*	7.97*E *−* *18	7.07*E *−* *18	7.07*E *−* *18	7.07*E *−* *18	1.45*E *−* *17
		*h*	*—*	1	1	1	1	1

*F*13	Mean		1.68*E* + 05	6.18*E* + 05	2.45*E* + 08	1.50*E* + 09	1.52*E* + 10	5.96*E* + 05
	Std.		7.11*E* + 04	3.89*E* + 05	3.01*E* + 08	5.85*E* + 08	2.51*E* + 09	5.72*E* + 05
	Time		3.27*E *−* *01	3.35*E *−* *01	2.93*E *−* *01	2.79*E* + 00	3.76*E *−* *01	1.47*E *−* *01
	Rank		1	3	4	5	6	2
	Test	*p*	*p*	1.47*E *−* *14	7.07*E *−* *18	7.07*E *−* *18	4.18*E *−* *18	7.27*E *−* *15
		*h*	*h*	1	1	1	1	1

*F*14	Mean		1.27*E* + 03	1.08*E* + 06	5.17*E* + 05	4.25*E* + 05	5.20*E* + 06	2.85*E* + 04
	Std.		2.68*E* + 03	2.25*E* + 05	3.48*E* + 05	1.36*E* + 05	1.00*E* + 06	3.10*E* + 04
	Time		5.59*E *−* *01	5.87*E *−* *01	4.73*E *−* *01	3.81*E* + 00	7.68*E *−* *01	3.19*E *−* *01
	Rank		1	5	4	3	6	2
	Test	*p*	*—*	7.07*E *−* *18	7.07*E *−* *18	7.07*E *−* *18	1.74*E *−* *18	2.27*E *−* *16
		*h*	*—*	1	1	1	1	1

*F*15	Mean		4.74*E* + 04	4.56*E* + 05	4.85*E* + 07	1.64*E* + 04	5.59*E* + 08	3.10*E* + 04
	Std.		3.30*E* + 04	5.69*E* + 05	3.17*E* + 07	5.05*E* + 03	1.94*E* + 08	1.48*E* + 04
	Time		3.08*E *−* *01	3.21*E *−* *01	2.76*E *−* *01	2.74*E* + 00	3.67*E *−* *01	1.39*E *−* *01
	Rank		3	4	5	1	6	2
	Test	*p*	*—*	1.85*E *−* *13	7.07*E *−* *18	1.6*E *−* *11	7.07*E *−* *18	0.002447
		*h*	*—*	1	1	1	1	1

*F*16	Mean		1.07*E* + 03	1.95*E* + 03	2.24*E* + 03	2.86*E* + 03	4.78*E* + 03	1.49*E* + 03
	Std.		2.98*E* + 02	3.84*E* + 02	3.06*E* + 02	4.39*E* + 02	5.79*E* + 02	2.75*E* + 02
	Time		5.02*E *−* *01	5.31*E *−* *01	4.19*E *−* *01	3.76*E* + 00	7.26*E *−* *01	2.92*E *−* *01
	Rank		1	3	4	5	6	2
	Test	*p*	*—*	7.67*E *−* *23	1.69*E *−* *35	7.73*E *−* *40	6.38*E *−* *18	6.98*E *−* *10
		*h*	*—*	1	1	1	1	1

*F*17	Mean		4.00*E* + 02	6.10*E* + 02	9.21*E* + 02	8.75*E* + 02	2.72*E* + 03	5.49*E* + 02
	Std.		1.94*E* + 02	2.68*E* + 02	2.93*E* + 02	2.37*E* + 02	4.84*E* + 02	1.88*E* + 02
	Time		7.17*E *−* *01	7.46*E *−* *01	6.38*E *−* *01	4.17*E* + 00	9.25*E *−* *01	4.05*E *−* *01
	Rank		1	3	5	4	6	2
	Test	*p*	*—*	5.12*E *−* *05	1.22*E *−* *13	8.05*E *−* *14	7.07*E *−* *18	0.000276
		*h*	*—*	1	1	1	1	1

*F*18	Mean		1.09*E* + 05	6.65*E* + 06	1.64*E* + 07	1.38*E* + 06	5.86*E* + 07	3.63*E* + 05
	Std.		6.86*E* + 04	4.25*E* + 06	6.02*E* + 06	1.02*E* + 06	2.94*E* + 07	3.08*E* + 05
	Time		5.05*E *−* *01	5.32*E *−* *01	4.19*E *−* *01	3.81*E* + 00	7.41*E *−* *01	2.90*E *−* *01
	Rank		1	4	5	3	6	2
	Test	*p*	*—*	7.07*E *−* *18	7.07*E *−* *18	2.47*E *−* *17	7.07*E *−* *18	8.95*E *−* *11
		*h*	*—*	1	1	1	1	1

*F*19	Mean		1.77*E* + 05	1.08*E* + 07	9.42*E* + 07	1.70*E* + 06	1.52*E* + 09	2.73*E* + 06
	Std.		2.08*E* + 05	5.81*E* + 06	6.88*E* + 07	1.21*E* + 06	5.26*E* + 08	1.59*E* + 06
	Time		1.67*E* + 00	1.69*E* + 00	1.58*E* + 00	5.02*E* + 00	1.77*E* + 00	9.66*E *−* *01
	Rank		1	3	5	4	6	2
	Test	*p*	*—*	7.07*E *−* *18	7.07*E *−* *18	1.6*E *−* *16	7.07*E *−* *18	5.02*E *−* *17
		*h*	*—*	1	1	1	1	1

*F*20	Mean		5.09*E* + 02	8.98*E* + 02	9.60*E* + 02	7.24*E* + 02	1.31*E* + 03	6.64*E* + 02
	Std.		1.31*E* + 02	5.75*E* + 01	1.22*E* + 02	1.65*E* + 02	1.25*E* + 02	8.64*E* + 01
	Time		7.81*E *−* *01	8.09*E *−* *01	6.89*E *−* *01	4.15*E* + 00	9.91*E *−* *01	6.07*E *−* *01
	Rank		1	4	5	3	6	2
	Test	*p*	*—*	1.37*E *−* *17	1.35*E *−* *32	9.87*E *−* *11	9.87*E *−* *11	6.13*E *−* *10
		*h*	*—*	1	1	1	1	1

Average rank			1.2	3.66	4.6	3.6	6	2
Overall rank			1	4	5	3	6	2

**Table 8 tab8:** The results of the algorithms on composition functions in *D* = 30.

Function			GMO	WOA	PSO	BRO	CSO	ABC
*F*21	Mean		3.31*E* + 02	4.82*E* + 02	5.21*E* + 02	5.34*E* + 02	6.96*E* + 02	3.64*E* + 02
	Std.		2.92*E* + 01	4.11*E* + 01	3.52*E* + 01	4.30*E* + 01	4.38*E* + 01	2.97*E* + 01
	Time		8.45*E *−* *01	8.77*E *−* *01	7.55*E *−* *01	4.14*E* + 00	1.08*E* + 00	5.28*E *−* *01
	Rank		1	3	4	5	6	2
	Test	*p*	*-*	2.01*E *−* *36	2.53*E *−* *50	1.63*E *−* *44	2.24*E *−* *64	1.73*E *−* *07
		*h*	*-*	1	1	1	1	1
*F*22	Mean		2.33*E* + 02	2.97*E* + 03	1.83*E* + 03	4.17*E* + 03	7.76*E* + 03	3.46*E* + 02
	Std.		8.90*E* + 02	2.20*E* + 03	4.37*E* + 02	6.19*E* + 02	6.63*E* + 02	7.91*E* + 01
	Time		7.19*E *−* *01	7.30*E *−* *01	6.88*E *−* *01	3.00*E* + 00	6.35*E *−* *01	3.36*E *−* *01
	Rank		1	4	3	5	6	2
	Test	*p*	*—*	1.13*E *−* *16	1.35*E *−* *16	1.35*E *−* *16	8.46*E *−* *18	1.43*E *−* *16
		*h*	*—*	1	1	1	1	1

*F*23	Mean		4.77*E* + 02	8.49*E* + 02	9.08*E* + 02	1.03*E* + 03	1.29*E* + 03	5.58*E* + 02
	Std.		4.03*E* + 01	9.40*E* + 01	8.77*E* + 01	6.95*E* + 01	1.04*E* + 02	4.27*E* + 01
	Time		9.90*E *−* *01	1.02*E* + 00	8.93*E *−* *01	4.22*E* + 00	1.14*E* + 00	6.21*E *−* *01
	Rank		1	3	4	5	6	2
	Test	*p*	*—*	7.07*E *−* *18	7.07*E *−* *18	7.07*E *−* *18	5.22*E *−* *18	8.97*E *−* *13
		*h*	*—*	1	1	1	1	1

*F*24	Mean		5.34*E* + 02	7.94*E* + 02	9.35*E* + 02	1.19*E* + 03	1.46*E* + 03	5.95*E* + 02
	Std.		3.28*E* + 01	7.54*E* + 01	3.68*E* + 01	9.28*E* + 01	1.32*E* + 02	3.78*E* + 01
	Time		1.07*E* + 00	1.09*E* + 00	9.61*E *−* *01	4.44*E* + 00	1.22*E* + 00	6.73*E *−* *01
	Rank		1	3	4	5	6	2
	Test	*p*	—	8.38*E *−* *33	2.35*E *−* *77	1.11*E *−* *49	1.19*E *−* *46	1.16*E *−* *13
		*h*	*—*	1	1	1	1	1

*F*25	Mean		4.28*E* + 02	5.65*E* + 02	1.31*E* + 03	9.64*E* + 02	3.21*E* + 03	5.60*E* + 02
	Std.		2.59*E* + 01	3.56*E* + 01	2.47*E* + 02	5.02*E* + 01	4.89*E* + 02	4.42*E* + 01
	Time		9.72*E *−* *01	9.96*E *−* *01	8.73*E *−* *01	4.26*E* + 00	1.17*E* + 00	5.67*E *−* *01
	Rank		1	3	5	4	6	2
	Test	*p*	*—*	5.82*E *−* *38	7.07*E *−* *18	1.39*E *−* *67	2.53*E *−* *39	6.16*E *−* *30
		*h*	*—*	1	1	1	1	1

*F*26	Mean		1.71*E* + 03	5.17*E* + 03	4.90*E* + 03	6.47*E* + 03	8.93*E* + 03	2.41*E* + 03
	Std.		1.14*E* + 03	8.86*E* + 02	5.86*E* + 02	4.56*E* + 02	5.11*E* + 02	1.66*E* + 03
	Time		1.17*E* + 00	1.20*E* + 00	1.08*E* + 00	4.45*E* + 00	1.29*E* + 00	7.06*E *−* *01
	Rank		1	4	3	5	6	2
	Test	*p*	*—*	7.07*E *−* *18	7.07*E *−* *18	7.07*E *−* *18	7.07*E *−* *18	0.021112
		*h*	*—*	1	1	1	1	1

*F*27	Mean		5.65*E* + 02	6.77*E* + 02	7.59*E* + 02	1.37*E* + 03	1.94*E* + 03	6.36*E* + 02
	Std.		2.54*E* + 01	4.73*E* + 01	4.28*E* + 01	1.72*E* + 02	2.25*E* + 02	6.65*E* + 01
	Time		1.31*E* + 00	1.34*E* + 00	1.20*E* + 00	4.61*E* + 00	1.47*E* + 00	7.89*E *−* *01
	Rank		1	3	4	5	6	2
	Test	*p*	*—*	3.13*E *−* *17	1.29*E *−* *42	7.07*E *−* *18	6.62*E *−* *18	1.26*E *−* *09
		*h*	*—*	1	1	1	1	1

*F*28	Mean		5.04*E* + 02	6.82*E* + 02	1.62*E* + 03	2.15*E* + 03	4.45*E* + 03	6.35*E* + 02
	Std.		2.78*E* + 01	6.03*E* + 01	4.84*E* + 02	1.67*E* + 02	4.89*E* + 02	5.99*E* + 01
	Time		1.16*E* + 00	1.18*E* + 00	1.05*E* + 00	4.47*E* + 00	1.32*E* + 00	6.76*E *−* *01
	Rank		1	3	4	5	6	2
	Test	*p*	*—*	7.07*E *−* *18	7.07*E *−* *18	7.07*E *−* *18	7.07*E *−* *18	3.74*E *−* *17
		*h*	*—*	1	1	1	1	1

*F*29	Mean		1.34*E* + 03	2.00*E* + 03	2.11*E* + 03	2.72*E* + 03	4.02*E* + 03	1.49*E* + 03
	Std.		2.13*E* + 02	4.41*E* + 02	4.51*E* + 02	4.45*E* + 02	5.05*E* + 02	2.77*E* + 02
	Time		1.00*E* + 00	1.03*E* + 00	9.06*E *−* *01	4.35*E* + 00	1.20*E* + 00	5.76*E *−* *01
	Rank		1	3	4	5	6	2
	Test	*p*	*—*	2.36*E *−* *14	1.13*E *−* *16	7.07*E *−* *18	5.12*E *−* *44	0.0026
		*h*	*—*	1	1	1	1	1

*F*30	Mean		1.89*E* + 06	2.27*E* + 07	1.07*E* + 08	6.80*E* + 07	2.75*E* + 09	1.06*E* + 07
	Std.		1.46*E* + 06	9.33*E* + 06	5.95*E* + 07	2.88*E* + 07	6.24*E* + 08	7.51*E* + 06
	Time		1.53*E* + 00	1.54*E* + 00	1.50*E* + 00	3.84*E* + 00	1.48*E* + 00	7.84*E *−* *01
	Rank		1	3	5	4	6	2
	Test	*p*	*—*	7.07*E *−* *18	7.07*E *−* *18	7.07*E *−* *18	4.98*E *−* *18	3.98*E *−* *15
		*h*	*—*	1	1	1	1	1

Average rank			1	3.2	4	4.8	6	2
Overall rank			1	3	4	5	6	2

**Table 9 tab9:** The results of the algorithms on unimodal and multimodal functions in *D* = 50.

Function			GMO	WOA	PSO	BRO	CSO	ABC
*F*1	Mean		7.42*E* + 05	4.17*E* + 09	4.27*E* + 10	7.02*E* + 10	1.15*E* + 11	8.38*E* + 09
	Std.		1.25*E* + 06	1.26*E* + 09	9.16*E* + 09	4.53*E* + 09	4.94*E* + 09	2.28*E* + 09
	Time		0.415468	0.429595	0.384812	2.886183	0.496381	0.199661
	Rank		1	2	4	5	6	3
	Test	*p*	*—*	7.07*E *−* *18	7.07*E *−* *18	7.07*E *−* *18	7.07*E *−* *18	7.07*E *−* *18
		*h*	*—*	1	1	1	1	1

*F*3	Mean		8.26*E* + 04	2.10*E* + 05	3.74*E* + 05	1.23*E* + 05	1.88*E* + 05	6.88*E* + 04
	Std.		2.28*E* + 04	6.38*E* + 04	7.47*E* + 04	8.71*E* + 03	1.71*E* + 04	1.06*E* + 04
	Time		4.17*E *−* *01	4.34*E *−* *01	3.94*E *−* *01	2.89*E* + 00	5.07*E *−* *01	2.01*E *−* *01
	Rank		2	4	6	3	5	1
	Test	*p*	*—*	8.46*E *−* *18	5.49*E *−* *34	2.12*E *−* *17	5.93*E *−* *44	2.29*E *−* *04
		*h*	*—*	1	1	1	1	1

*F*4	Mean		2.87*E* + 02	1.11*E* + 03	5.54*E* + 03	1.74*E* + 04	3.59*E* + 04	1.41*E* + 03
	Std.		4.70*E* + 01	2.38*E* + 02	2.42*E* + 03	1.41*E* + 03	3.54*E* + 03	4.33*E* + 02
	Time		4.12*E *−* *01	4.21*E *−* *01	3.85*E *−* *01	2.88*E* + 00	4.90*E *−* *01	1.97*E *−* *01
	Rank		1	2	4	5	6	3
	Test	*p*	*—*	3.63*E *−* *30	7.07*E *−* *18	3.16*E *−* *55	4.06*E *−* *51	1.02*E *−* *23
		*h*	*—*	1	1	1	1	1

*F*5	Mean		2.85*E* + 02	5.36*E* + 02	6.78*E* + 02	5.60*E* + 02	7.57*E* + 02	4.34*E* + 02
	Std.		4.54*E* + 01	9.10*E* + 01	4.72*E* + 01	4.94*E* + 01	1.88*E* + 01	5.18*E* + 01
	Time		5.25*E *−* *01	5.42*E *−* *01	4.99*E *−* *01	2.98*E* + 00	5.55*E *−* *01	2.57*E *−* *01
	Rank		1	3	5	4	6	2
	Test	*p*	*—*	1.35*E *−* *27	7.60*E *−* *65	1.35*E *−* *27	2.51*E *−* *62	1.06*E *−* *27
		*h*	*—*	1	1	1	1	1

*F*6	Mean		4.65*E* + 01	8.57*E* + 01	7.95*E* + 01	8.71*E* + 01	1.05*E* + 02	5.42*E* + 01
	Std.		8.74*E* + 00	8.46*E* + 00	1.00*E* + 01	7.95*E* + 00	2.80*E* + 00	9.16*E* + 00
	Time		8.37*E *−* *01	8.48*E *−* *01	8.07*E *−* *01	3.29*E* + 00	9.39*E *−* *01	4.20*E *−* *01
	Rank		1	4	3	5	6	2
	Test	*p*	*—*	5.13*E *−* *41	4.38*E *−* *32	2.43*E *−* *43	1.34*E *−* *47	3.48*E *−* *05
		*h*	*—*	1	1	1	1	1

*F*7	Mean		4.45*E* + 02	1.11*E* + 03	1.50*E* + 03	1.04*E* + 03	1.38*E* + 03	7.10*E* + 02
	Std.		1.07*E* + 02	9.64*E* + 01	1.81*E* + 02	7.93*E* + 01	2.29*E* + 01	5.52*E* + 01
	Time		5.39*E *−* *01	5.57*E *−* *01	5.10*E *−* *01	2.99*E* + 00	5.71*E *−* *01	2.63*E *−* *01
	Rank		1	4	6	3	5	2
	Test	*p*	*—*	7.5*E *−* *18	7.07*E *−* *18	8.46*E *−* *18	7.07*E *−* *18	9.92*E *−* *16
		*h*	*—*	1	1	1	1	1

*F*8	Mean		3.08*E* + 02	5.41*E* + 02	6.68*E* + 02	5.91*E* + 02	7.90*E* + 02	4.63*E* + 02
	Std.		6.71*E* + 01	8.78*E* + 01	5.30*E* + 01	3.96*E* + 01	1.93*E* + 01	4.23*E* + 01
	Time		5.39*E *−* *01	5.53*E *−* *01	5.13*E *−* *01	3.03*E* + 00	5.60*E *−* *01	2.64*E *−* *01
	Rank		1	3	5	4	6	2
	Test	*p*	*—*	4.21*E *−* *17	8.00*E *−* *51	3.84*E *−* *40	7.07*E *−* *18	3.99*E *−* *23
		*h*	*—*	1	1	1	1	1

*F*9	Mean		1.14*E* + 04	2.61*E* + 04	3.44*E* + 04	2.66*E* + 04	4.66*E* + 04	1.34*E* + 04
	Std.		4.58*E* + 03	5.24*E* + 03	7.17*E* + 03	4.95*E* + 03	2.43*E* + 03	4.83*E* + 03
	Time		5.35*E *−* *01	5.45*E *−* *01	5.06*E *−* *01	2.98*E* + 00	5.60*E *−* *01	2.63*E *−* *01
	Rank		1	3	5	4	6	2
	Test	*p*	*—*	2.54*E *−* *16	1.73*E *−* *17	1.6*E *−* *16	7.07*E *−* *18	0.017883
		*h*	*—*	1	1	1	1	1

*F*10	Mean		9.31*E* + 03	1.03*E* + 04	1.37*E* + 04	1.32*E* + 04	1.52*E* + 04	1.38*E* + 04
	Std.		1.54*E* + 03	6.01*E* + 02	7.92*E* + 02	9.00*E* + 02	4.26*E* + 02	3.68*E* + 02
	Time		6.01*E *−* *01	6.12*E *−* *01	5.82*E *−* *01	3.05*E* + 00	6.05*E *−* *01	3.03*E *−* *01
	Rank		1	2	4	3	6	5
	Test	*p*	*—*	1.52*E *−* *05	3.13*E *−* *17	2.54*E *−* *16	4.45*E *−* *18	7.97*E *−* *18
		*h*	*—*	1	1	1	1	1

Average rank			1.1	3	4.67	4	5.78	2.44
Overall rank			4	6	5	4	6	5

**Table 10 tab10:** The results of the algorithms on hybrid functions in *D* = 50.

Function			GMO	WOA	PSO	BRO	CSO	ABC
*F*11	Mean		5.94*E* + 02	2.03*E* + 03	1.51*E* + 04	9.54*E* + 03	2.27*E* + 04	2.02*E* + 03
	Std.		1.42*E* + 02	6.84*E* + 02	4.28*E* + 03	1.21*E* + 03	2.46*E* + 03	5.01*E* + 02
	Time		4.77*E *−* *01	4.86*E *−* *01	4.49*E *−* *01	2.95*E* + 00	5.46*E *−* *01	2.27*E *−* *01
	Rank		1	3	5	4	6	2
	Test	*p*	*—*	7.07*E *−* *18	7.07*E *−* *18	7.07*E *−* *18	7.07*E *−* *18	7.07*E *−* *18
		*h*	*—*	1	1	1	1	1

*F*12	Mean		2.18*E* + 08	8.46*E* + 08	1.16*E* + 10	4.07*E* + 10	8.87*E* + 10	1.07*E* + 09
	Std.		1.35*E* + 08	3.38*E* + 08	4.24*E* + 09	7.04*E* + 09	8.91*E* + 09	4.97*E* + 08
	Time		5.36*E *−* *01	5.53*E *−* *01	5.12*E *−* *01	3.03*E* + 00	6.03*E *−* *01	2.61*E *−* *01
	Rank		1	2	4	5	6	3
	Test	*p*	*—*	5.97*E *−* *16	7.07*E *−* *18	7.07*E *−* *18	7.07*E *−* *18	1.6*E *−* *16
		*h*	*—*	1	1	1	1	1

*F*13	Mean		1.60*E* + 05	3.82*E* + 07	7.08*E* + 09	1.36*E* + 10	5.46*E* + 10	4.54*E* + 07
	Std.		6.51*E* + 04	3.32*E* + 07	7.48*E* + 09	3.63*E* + 09	8.80*E* + 09	3.06*E* + 07
	Time		4.71*E *−* *01	4.79*E *−* *01	4.39*E *−* *01	2.93*E* + 00	5.27*E *−* *01	2.26*E *−* *01
	Rank		1	2	4	5	6	3
	Test	*p*	*—*	7.07*E *−* *18	7.07*E *−* *18	1.03*E *−* *30	5.45*E *−* *41	7.07*E *−* *18
		*h*	*—*	1	1	1	1	1

*F*14	Mean		8.87*E* + 04	3.46*E* + 06	2.26*E* + 07	1.14*E* + 07	9.15*E* + 07	6.50*E* + 05
	Std.		6.95*E* + 04	1.78*E* + 06	1.49*E* + 07	5.78*E* + 06	3.62*E* + 07	4.69*E* + 05
	Time		6.06*E *−* *01	6.19*E *−* *01	5.68*E *−* *01	3.05*E* + 00	6.29*E *−* *01	2.95*E *−* *01
	Rank		1	3	5	4	6	2
	Test	*p*	*—*	7.07*E *−* *18	7.07*E *−* *18	7.07*E *−* *18	7.06*E *−* *18	2.17*E *−* *15
		*h*	*—*	1	1	1	1	1

*F*15	Mean		4.47*E* + 04	7.27*E* + 06	1.57*E* + 09	1.19*E* + 09	6.61*E* + 09	7.56*E* + 05
	Std.		2.76*E* + 04	6.66*E* + 06	1.92*E* + 09	4.28*E* + 08	1.33*E* + 09	4.19*E* + 05
	Time		4.59*E *−* *01	4.71*E *−* *01	4.31*E *−* *01	2.96*E* + 00	5.34*E *−* *01	2.20*E *−* *01
	Rank		1	3	5	4	6	2
	Test	*p*	*—*	7.07*E *−* *18	7.07*E *−* *18	7.07*E *−* *18	7.07*E *−* *18	7.07*E *−* *18
		*h*	*—*	1	1	1	1	1

*F*16	Mean		2.03*E* + 03	3.86*E* + 03	4.40*E* + 03	5.19*E* + 03	8.49*E* + 03	2.63*E* + 03
	Std.		4.29*E* + 02	6.87*E* + 02	4.93*E* + 02	6.73*E* + 02	1.19*E* + 03	4.15*E* + 02
	Time		5.12*E *−* *01	5.28*E *−* *01	4.87*E *−* *01	2.98*E* + 00	5.76*E *−* *01	2.48*E *−* *01
	Rank		1	3	4	5	6	2
	Test	*p*	*—*	5.21*E *−* *27	2.40*E *−* *45	7.07*E *−* *18	4.57*E *−* *43	1.35*E *−* *10
		*h*	*—*	1	1	1	1	1

*F*17	Mean		1.87*E* + 03	2.61*E* + 03	3.57*E* + 03	2.75*E* + 03	1.34*E* + 04	1.82*E* + 03
	Std.		3.36*E* + 02	4.02*E* + 02	3.40*E* + 02	4.20*E* + 02	4.46*E* + 03	2.63*E* + 02
	Time		7.72*E *−* *01	7.79*E *−* *01	7.48*E *−* *01	3.27*E* + 00	7.78*E *−* *01	3.78*E *−* *01
	Rank		2	3	5	4	6	1
	Test	*p*	*—*	1.44*E *−* *16	1.46*E *−* *44	3.53*E *−* *20	1.48*E *−* *23	0.4231
		*h*	*—*	1	1	1	1	0

*F*18	Mean		1.03*E* + 06	1.70*E* + 07	3.66*E* + 07	2.59*E* + 07	2.18*E* + 08	3.77*E* + 06
	Std.		1.02*E* + 06	6.60*E* + 06	1.30*E* + 07	6.18*E* + 06	6.86*E* + 07	2.32*E* + 06
	Time		5.02*E *−* *01	5.19*E *−* *01	4.76*E *−* *01	2.95*E* + 00	5.75*E *−* *01	2.43*E *−* *01
	Rank		1	3	5	4	6	2
	Test	*p*	*—*	7.07*E *−* *18	7.07*E *−* *18	7.07*E *−* *18	7.07*E *−* *18	7.8*E *−* *12
		*h*	*—*	1	1	1	1	1

*F*19	Mean		2.68*E* + 05	1.69*E* + 06	2.58*E* + 08	4.68*E* + 08	6.74*E* + 09	5.38*E* + 06
	Std.		2.69*E* + 05	2.06*E* + 06	1.17*E* + 08	2.18*E* + 08	1.13*E* + 09	5.33*E* + 06
	Time		2.08*E* + 00	2.09*E* + 00	2.04*E* + 00	4.53*E* + 00	1.94*E* + 00	1.06*E* + 00
	Rank		1	2	4	5	6	3
	Test	*p*	*—*	6.13*E *−* *12	7.07*E *−* *18	7.07*E *−* *18	7.07*E *−* *18	9.53*E *−* *17
		*h*	*—*	1	1	1	1	1

*F*20	Mean		1.31*E* + 03	1.75*E* + 03	2.16*E* + 03	1.64*E* + 03	2.65*E* + 03	1.84*E* + 03
	Std.		2.96*E* + 02	3.31*E* + 02	1.96*E* + 02	3.38*E* + 02	1.53*E* + 02	2.20*E* + 02
	Time		8.16*E *−* *01	8.30*E *−* *01	7.95*E *−* *01	3.32*E* + 00	8.10*E *−* *01	4.12*E *−* *01
	Rank		1	3	5	2	6	4
	Test	*p*	*—*	6.59*E *−* *09	7.79*E *−* *29	1.15*E *−* *06	2.50*E *−* *41	1.53*E *−* *16
		*h*	*—*	1	1	1	1	1

Average rank			1.1	2.7	4.6	4.2	6	2.4
Overall rank			1	3	5	4	6	2

**Table 11 tab11:** The results of the algorithms on composition functions in *D* = 50.

Function			GMO	WOA	PSO	BRO	CSO	ABC
*F*21	Mean		4.78*E* + 02	8.69*E* + 02	8.70*E* + 02	8.94*E* + 02	1.19*E* + 03	6.15*E* + 02
	Std.		5.83*E* + 01	9.85*E* + 01	5.51*E* + 01	6.31*E* + 01	5.33*E* + 01	6.66*E* + 01
	Time		1.15*E* + 00	1.17*E* + 00	1.12*E* + 00	3.62*E* + 00	1.19*E* + 00	5.85*E *−* *01
	Rank		1	3	4	5	6	2
	Test	*p*	*—*	2.15*E *−* *38	1.16*E *−* *56	2.76*E *−* *56	1.10*E *−* *81	1.25*E *−* *18
		*h*	*—*	1	1	1	1	1

*F*22	Mean		9.00*E* + 03	1.19*E* + 04	1.38*E* + 04	1.37*E* + 04	1.60*E* + 04	4.10*E* + 03
	Std.		1.53*E* + 03	1.00*E* + 03	8.51*E* + 02	8.58*E* + 02	2.40*E* + 02	3.34*E* + 03
	Time		1.29*E* + 00	1.30*E* + 00	1.26*E* + 00	3.76*E* + 00	1.17*E* + 00	6.58*E *−* *01
	Rank		2	3	5	4	6	1
	Test	*p*	*—*	1.00*E *−* *18	3.13*E *−* *17	6.90*E *−* *31	2.49*E *−* *18	1.26*E *−* *11
		*h*	*—*	1	1	1	1	1

*F*23	Mean		7.31*E* + 02	1.43*E* + 03	1.50*E* + 03	1.90*E* + 03	2.30*E* + 03	1.04*E* + 03
	Std.		6.55*E* + 01	1.51*E* + 02	1.68*E* + 02	1.54*E* + 02	1.31*E* + 02	7.27*E* + 01
	Time		1.49*E* + 00	1.50*E* + 00	1.45*E* + 00	4.01*E* + 00	1.39*E* + 00	7.61*E *−* *01
	Rank		1	3	4	5	6	2
	Test	*p*	*—*	1.78*E *−* *40	1.47*E *−* *39	8.41*E *−* *54	5.87*E *−* *18	8.44*E *−* *40
		*h*	*—*	1	1	1	1	1

*F*24	Mean		7.88*E* + 02	1.42*E* + 03	1.58*E* + 03	2.15*E* + 03	2.68*E* + 03	1.05*E* + 03
	Std.		5.84*E* + 01	1.58*E* + 02	9.94*E* + 01	1.38*E* + 02	2.75*E* + 02	6.91*E* + 01
	Time		1.61*E* + 00	1.62*E* + 00	1.55*E* + 00	4.09*E* + 00	1.52*E* + 00	8.20*E *−* *01
	Rank		1	3	4	5	6	2
	Test	*p*	*—*	1.06*E *−* *35	7.07*E *−* *18	2.97*E *−* *61	6.72*E *−* *18	2.50*E *−* *37
		*h*	*—*	1	1	1	1	1

*F*25	Mean		6.85*E* + 02	1.26*E* + 03	6.57*E* + 03	6.91*E* + 03	1.37*E* + 04	1.50*E* + 03
	Std.		5.39*E* + 01	1.98*E* + 02	1.99*E* + 03	4.82*E* + 02	6.50*E* + 02	2.83*E* + 02
	Time		1.53*E* + 00	1.54*E* + 00	1.49*E* + 00	3.96*E* + 00	1.51*E* + 00	7.74*E *−* *01
	Rank		1	2	4	5	6	3
	Test	*p*	*—*	4.20*E *−* *27	4.25*E *−* *26	2.45*E *−* *57	2.54*E *−* *66	2.43*E *−* *26
		*h*	*—*	1	1	1	1	1

*F*26	Mean		4.28*E* + 03	1.16*E* + 04	9.70*E* + 03	1.15*E* + 04	1.52*E* + 04	6.33*E* + 03
	Std.		1.16*E* + 03	1.53*E* + 03	1.69*E* + 03	6.21*E* + 02	5.18*E* + 02	2.60*E* + 03
	Time		1.84*E* + 00	1.85*E* + 00	1.81*E* + 00	4.30*E* + 00	1.70*E* + 00	9.40*E *−* *01
	Rank		1	5	3	4	6	2
	Test	*P*	*—*	7.07*E *−* *18	1.14*E *−* *17	7.07*E *−* *18	7.07*E *−* *18	0.000492
		*H*	*—*	1	1	1	1	1

*F*27	Mean		9.50*E* + 02	2.09*E* + 03	1.88*E* + 03	3.54*E* + 03	5.20*E* + 03	1.37*E* + 03
	Std.		1.55*E* + 02	3.23*E* + 02	1.95*E* + 02	2.85*E* + 02	5.77*E* + 02	2.30*E* + 02
	Time		2.10*E* + 00	2.11*E* + 00	2.04*E* + 00	4.58*E* + 00	2.04*E* + 00	1.08*E* + 00
	Rank		1	4	3	5	6	2
	Test	*p*	*—*	7.07*E *−* *18	1.82*E *−* *46	1.29*E *−* *63	2.39*E *−* *48	2.81*E *−* *17
		*h*	*—*	1	1	1	1	1

*F*28	Mean		7.80*E* + 02	1.88*E* + 03	4.48*E* + 03	6.09*E* + 03	1.12*E* + 04	2.14*E* + 03
	Std.		1.26*E* + 02	3.13*E* + 02	1.03*E* + 03	3.45*E* + 02	7.31*E* + 02	3.32*E* + 02
	Time		1.88*E* + 00	1.90*E* + 00	1.84*E* + 00	4.34*E* + 00	1.82*E* + 00	9.63*E *−* *01
	Rank		1	2	4	5	6	3
	Test	*p*	*—*	6.44*E *−* *33	3.66*E *−* *30	1.03*E *−* *70	7.98*E *−* *61	2.35*E *−* *36
		*h*	*—*	1	1	1	1	1

*F*29	Mean		2.43*E* + 03	4.90*E* + 03	5.53*E* + 03	1.01*E* + 04	4.36*E* + 04	3.38*E* + 03
	Std.		4.26*E* + 02	8.28*E* + 02	1.79*E* + 03	1.93*E* + 03	1.78*E* + 04	5.21*E* + 02
	Time		1.35*E* + 00	1.36*E* + 00	1.31*E* + 00	3.86*E* + 00	1.32*E* + 00	6.78*E *−* *01
	Rank		1	3	4	5	6	2
	Test	*p*	*—*	7.07*E *−* *18	8.46*E *−* *18	7.07*E *−* *18	7.07*E *−* *18	8.53*E *−* *13
		*h*	*—*	1	1	1	1	1

*F*30	Mean		4.63*E* + 07	3.68*E* + 08	7.90*E* + 08	1.16*E* + 09	9.51*E* + 09	2.42*E* + 08
	Std.		1.53*E* + 07	8.18*E* + 07	3.99*E* + 08	3.11*E* + 08	1.26*E* + 09	5.05*E* + 07
	Time		2.70*E* + 00	2.71*E* + 00	2.66*E* + 00	5.22*E* + 00	2.54*E* + 00	1.38*E* + 00
	Rank		1	3	4	5	6	2
	Test	*p*	*—*	7.07*E *−* *18	7.07*E *−* *18	7.07*E *−* *18	7.06*E *−* *18	7.07*E *−* *18
		*h*	*—*	1	1	1	1	1

Average rank			1.1	3.1	3.9	4.8	6	2.1
Overall rank			1	3	4	5	6	2

**Table 12 tab12:** The results of the algorithms on unimodal and multimodal functions in *D* = 100.

Function			GMO	WOA	PSO	BRO	CSO	ABC
*F*1	Mean		1.11*E* + 10	4.66*E* + 10	2.08*E* + 11	2.27*E* + 11	2.73*E* + 11	6.53*E* + 10
	Std.		3.00*E* + 09	7.43*E* + 09	2.73*E* + 10	6.41*E* + 09	4.77*E* + 09	9.38*E* + 09
	Time		1.32*E* + 00	1.36*E* + 00	1.34*E* + 00	4.25*E* + 00	1.39*E* + 00	6.54*E *−* *01
	Rank		1	2	4	5	6	3
	Test	*p*	*—*	9.24*E *−* *41	9.02*E *−* *45	4.39*E *−* *100	1.81*E *−* *130	9.28*E *−* *44
		*h*	*—*	1	1	1	1	1

*F*3	Mean		3.99*E* + 05	8.64*E* + 05	8.70*E* + 05	3.22*E* + 05	3.77*E* + 05	2.57*E* + 05
	Std.		7.36*E* + 04	5.37*E* + 04	1.74*E* + 05	1.77*E* + 04	2.71*E* + 04	2.14*E* + 04
	Time		1.33*E* + 00	1.37*E* + 00	1.35*E* + 00	4.36*E* + 00	1.41*E* + 00	6.55*E *−* *01
	Rank		4	5	6	2	3	1
	Test	*p*	*—*	7.07*E *−* *18	1.17*E *−* *26	4.01*E *−* *09	0.191424	6.25*E *−* *19
		*h*	*—*	1	1	1	0	1

*F*4	Mean		1.69*E* + 03	8.28*E* + 03	4.13*E* + 04	5.71*E* + 04	1.06*E* + 05	1.04*E* + 04
	Std.		4.29*E* + 02	1.37*E* + 03	8.15*E* + 03	4.02*E* + 03	7.40*E* + 03	1.68*E* + 03
	Time		1.33*E* + 00	1.37*E* + 00	1.35*E* + 00	4.39*E* + 00	1.41*E* + 00	6.58*E *−* *01
	Rank		1	2	4	5	6	3
	Test	*p*	*—*	7.07*E *−* *18	7.07*E *−* *18	7.07*E *−* *18	7.07*E *−* *18	7.07*E *−* *18
		*h*	*—*	1	1	1	1	1

*F*5	Mean		9.49*E* + 02	1.29*E* + 03	1.66*E* + 03	1.52*E* + 03	1.69*E* + 03	1.18*E* + 03
	Std.		1.03*E* + 02	1.40*E* + 02	9.75*E* + 01	5.35*E* + 01	2.84*E* + 01	6.18*E* + 01
	Time		1.53*E* + 00	1.58*E* + 00	1.55*E* + 00	4.55*E* + 00	1.50*E* + 00	7.68*E *−* *01
	Rank		1	3	5	4	6	2
	Test	*p*	*—*	5.38*E *−* *24	8.63*E *−* *58	1.27*E *−* *47	6.29*E *−* *48	1.04*E *−* *22
		*h*	*—*	1	1	1	1	1

*F*6	Mean		6.98*E* + 01	9.93*E* + 01	1.11*E* + 02	1.07*E* + 02	1.14*E* + 02	8.55*E* + 01
	Std.		7.09*E* + 00	8.33*E* + 00	6.61*E* + 00	4.37*E* + 00	2.96*E* + 00	6.97*E* + 00
	Time		2.15*E* + 00	2.19*E* + 00	2.17*E* + 00	5.20*E* + 00	2.26*E* + 00	1.10*E* + 00
	Rank		1	3	5	4	6	2
	Test	*p*	—	8.66*E *−* *35	7.07*E *−* *18	2.94*E *−* *47	1.66*E *−* *48	3.55*E *−* *19
		*h*	*—*	1	1	1	1	1

*F*7	Mean		1.68*E* + 03	2.76*E* + 03	3.78*E* + 03	2.83*E* + 03	3.41*E* + 03	2.11*E* + 03
	Std.		2.67*E* + 02	1.39*E* + 02	3.84*E* + 02	1.34*E* + 02	6.48*E* + 01	1.38*E* + 02
	Time		1.56*E* + 00	1.60*E* + 00	1.57*E* + 00	4.59*E* + 00	1.52*E* + 00	7.82*E *−* *01
	Rank		1	3	6	4	5	2
	Test	*p*	*—*	2.73*E *−* *38	7.26*E *−* *50	8.50*E *−* *40	7.07*E *−* *18	1.56*E *−* *15
		*h*	*—*	1	1	1	1	1

*F*8	Mean		9.74*E* + 02	1.45*E* + 03	1.72*E* + 03	1.66*E* + 03	1.87*E* + 03	1.28*E* + 03
	Std.		1.07*E* + 02	1.20*E* + 02	9.64*E* + 01	6.52*E* + 01	2.85*E* + 01	8.21*E* + 01
	Time		1.56*E* + 00	1.60*E* + 00	1.58*E* + 00	4.59*E* + 00	1.51*E* + 00	7.83*E *−* *01
	Rank		1	3	5	4	6	2
	Test	*p*	*—*	3.39*E *−* *38	3.67*E *−* *59	6.51*E *−* *54	2.17*E *−* *51	1.20*E *−* *29
		*h*	*—*	1	1	1	1	1

*F*9	Mean		4.29*E* + 04	6.79*E* + 04	1.06*E* + 05	7.42*E* + 04	8.91*E* + 04	6.46*E* + 04
	Std.		9.88*E* + 03	1.70*E* + 04	1.64*E* + 04	1.18*E* + 04	3.14*E* + 03	5.49*E* + 03
	Time		1.57*E* + 00	1.61*E* + 00	1.58*E* + 00	4.56*E* + 00	1.53*E* + 00	7.90*E *−* *01
	Rank		1	3	6	4	5	2
	Test	*p*	*—*	9.70*E *−* *14	7.07*E *−* *18	1.01*E *−* *16	1.55*E *−* *38	4.00*E *−* *22
		*h*	*—*	1	1	1	1	1

*F*10	Mean		2.13*E* + 04	2.48*E* + 04	3.09*E* + 04	2.97*E* + 04	3.24*E* + 04	3.09*E* + 04
	Std.		2.88*E* + 03	1.64*E* + 03	1.09*E* + 03	1.19*E* + 03	5.27*E* + 02	6.00*E* + 02
	Time		1.70*E* + 00	1.74*E* + 00	1.73*E* + 00	4.72*E* + 00	1.61*E* + 00	8.70*E *−* *01
	Rank		1	2	5	3	6	4
	Test	*p*	*—*	1.01*E *−* *10	8.46*E *−* *18	1.92*E *−* *28	7.07*E *−* *18	7.07*E *−* *18
		*h*	*—*	1	1	1	1	1

Average rank			1.33	2.89	5.11	3.89	5.44	2.33
Overall rank			1	3	5	4	6	2

**Table 13 tab13:** The results of the algorithms on hybrid functions in *D* = 100.

Function			GMO	WOA	PSO	BRO	CSO	ABC
*F*11	Mean		3.46*E* + 04	2.47*E* + 05	2.91*E* + 05	1.33*E* + 05	2.19*E* + 05	5.46*E* + 04
	Std.		1.19*E* + 04	6.86*E* + 04	6.59*E* + 04	1.34*E* + 04	3.24*E* + 04	9.89*E* + 03
	Time		1.38*E* + 00	1.42*E* + 00	1.43*E* + 00	4.31*E* + 00	1.42*E* + 00	6.82*E *−* *01
	Rank		1	5	6	3	4	2
	Test	*p*	*—*	7.07*E *−* *18	7.07*E *−* *18	7.07*E *−* *18	7.07*E *−* *18	1.52*E *−* *11
		*h*	*—*	1	1	1	1	1

*F*12	Mean		1.19*E* + 09	7.02*E* + 09	5.35*E* + 10	1.52*E* + 11	2.13*E* + 11	1.17*E* + 10
	Std.		4.83*E* + 08	1.68*E* + 09	1.30*E* + 10	8.02*E* + 09	1.07*E* + 10	2.61*E* + 09
	Time		1.56*E* + 00	1.60*E* + 00	1.59*E* + 00	4.62*E* + 00	1.60*E* + 00	7.79*E *−* *01
	Rank		1	2	4	5	6	3
	Test	*p*	*—*	4.65*E *−* *31	3.78*E *−* *32	7.07*E *−* *18	7.07*E *−* *18	2.73*E *−* *33
		*h*	*—*	1	1	1	1	1

*F*13	Mean		7.06*E* + 04	1.81*E* + 08	7.45*E* + 09	2.98*E* + 10	5.08*E* + 10	3.83*E* + 08
	Std.		2.61*E* + 04	9.61*E* + 07	2.38*E* + 09	2.28*E* + 09	3.71*E* + 09	1.29*E* + 08
	Time		1.44*E* + 00	1.48*E* + 00	1.46*E* + 00	4.50*E* + 00	1.48*E* + 00	7.17*E *−* *01
	Rank		1	2	4	5	6	3
	Test	*p*	*—*	7.22*E *−* *18	3.52*E *−* *27	1.16*E *−* *56	1.36*E *−* *57	4.69*E *−* *26
		*h*	*—*	1	1	1	1	1

*F*14	Mean		1.75*E* + 06	9.61*E* + 06	3.97*E* + 07	2.50*E* + 07	1.46*E* + 08	6.46*E* + 06
	Std.		9.23*E* + 05	3.00*E* + 06	1.52*E* + 07	8.26*E* + 06	3.88*E* + 07	2.43*E* + 06
	Time		1.66*E* + 00	1.69*E* + 00	1.69*E* + 00	4.58*E* + 00	1.60*E* + 00	8.23*E *−* *01
	Rank		1	3	5	4	6	2
	Test	*p*	*—*	4.15*E *−* *25	6.07*E *−* *23	7.07*E *−* *18	1.65*E *−* *30	3.93*E *−* *19
		*h*	*—*	1	1	1	1	1

*F*15	Mean		7.08*E* + 04	2.45*E* + 07	3.93*E* + 09	1.27*E* + 10	2.96*E* + 10	2.58*E* + 07
	Std.		2.83*E* + 04	2.38*E* + 07	1.74*E* + 09	2.84*E* + 09	2.05*E* + 09	1.17*E* + 07
	Time		1.36*E* + 00	1.40*E* + 00	1.41*E* + 00	4.29*E* + 00	1.41*E* + 00	6.73*E *−* *01
	Rank		1	2	4	5	6	3
	Test	*p*	*—*	7.07*E *−* *18	4.88*E *−* *21	2.48*E *−* *34	9.79*E *−* *59	7.07*E *−* *18
		*h*	*—*	1	1	1	1	1

*F*16	Mean		6.14*E* + 03	1.57*E* + 04	1.25*E* + 04	1.64*E* + 04	2.33*E* + 04	8.37*E* + 03
	Std.		8.04*E* + 02	2.14*E* + 03	1.12*E* + 03	1.66*E* + 03	1.81*E* + 03	1.04*E* + 03
	Time		1.52*E* + 00	1.56*E* + 00	1.54*E* + 00	4.60*E* + 00	1.54*E* + 00	7.56*E *−* *01
	Rank		1	4	3	5	6	2
	Test	*p*	*—*	2.57*E *−* *09	4.88*E *−* *21	2.48*E *−* *34	9.79*E *−* *59	5.64*E *−* *16
		*h*	*—*	1	1	1	1	1

*F*17	Mean		3.99*E* + 03	6.17*E* + 03	1.87*E* + 04	3.22*E* + 05	7.03*E* + 06	5.07*E* + 03
	Std.		5.11*E* + 02	1.06*E* + 03	1.28*E* + 04	1.67*E* + 05	2.75*E* + 06	6.51*E* + 02
	Time		1.94*E* + 00	1.97*E* + 00	2.00*E* + 00	4.89*E* + 00	1.85*E* + 00	9.74*E *−* *01
	Rank		1	3	4	5	6	2
	Test	*p*	*—*	1.2*E *−* *16	7.07*E *−* *18	7.07*E *−* *18	6.98*E *−* *18	4.36*E *−* *12
		*h*	*—*	1	1	1	1	1

*F*18	Mean		2.05*E* + 06	9.42*E* + 06	5.24*E* + 07	6.44*E* + 07	2.55*E* + 08	7.62*E* + 06
	Std.		1.31*E* + 06	3.41*E* + 06	1.74*E* + 07	2.20*E* + 07	6.96*E* + 07	3.09*E* + 06
	Time		1.52*E* + 00	1.56*E* + 00	1.53*E* + 00	4.49*E* + 00	1.56*E* + 00	7.53*E *−* *01
	Rank		1	3	4	5	6	2
	Test	*p*	*—*	3.32*E *−* *17	7.07*E *−* *18	7.07*E *−* *18	7.07*E *−* *18	3.58*E *−* *16
		*h*	*—*	1	1	1	1	1

*F*19	Mean		1.14*E* + 07	4.74*E* + 07	3.33*E* + 09	1.19*E* + 10	2.82*E* + 10	3.68*E* + 07
	Std.		8.24*E* + 06	3.10*E* + 07	1.12*E* + 09	1.33*E* + 09	2.70*E* + 09	2.66*E* + 07
	Time		4.63*E* + 00	4.67*E* + 00	4.68*E* + 00	7.62*E* + 00	4.29*E* + 00	2.36*E* + 00
	Rank		1	3	5	4	6	2
	Test	*p*	*—*	2.52*E *−* *13	3.96*E *−* *26	1.21*E *−* *48	6.03*E *−* *52	5.05*E *−* *12
		*h*	*—*	1	1	1	1	1

*F*20	Mean		3.76*E* + 03	4.80*E* + 03	6.23*E* + 03	5.22*E* + 03	6.38*E* + 03	5.42*E* + 03
	Std.		5.48*E* + 02	4.45*E* + 02	3.40*E* + 02	4.90*E* + 02	2.78*E* + 02	2.86*E* + 02
	Time		2.10*E* + 00	2.14*E* + 00	2.13*E* + 00	5.19*E* + 00	1.97*E* + 00	1.08*E* + 00
	Rank		1	2	5	3	6	4
	Test	*p*	*—*	2.01*E *−* *17	7.5*E *−* *18	1.39*E *−* *15	8.52*E *−* *43	5.02*E *−* *0
		*h*	*—*	1	1	1	1	1

Average rank			1	2.9	4.4	4.4	5.8	2.5
Overall rank			1	3	4	4	6	2

**Table 14:** The results of the algorithms on composition functions in *D* = 100.

Function			GMO	WOA	PSO	BRO	CSO	ABC
*F*21	Mean		1.13*E* + 03	2.08*E* + 03	2.09*E* + 03	2.34*E* + 03	3.03*E* + 03	1.54*E* + 03
	Std.		1.27*E* + 02	1.52*E* + 02	1.01*E* + 02	1.14*E* + 02	1.27*E* + 02	1.04*E* + 02
	Time		4.06*E* + 00	4.11*E* + 00	4.08*E* + 00	7.07*E* + 00	4.04*E* + 00	2.09*E* + 00
	Rank		1	3	4	5	6	2
	Test	*p*	*—*	7.07*E *−* *18	2.70*E *−* *64	1.07*E *−* *71	6.72*E *−* *18	6.56E-32
		*h*	*—*	1	1	1	1	1

*F*22	Mean		2.37*E* + 04	2.72*E* + 04	3.14*E* + 04	3.12*E* + 04	3.43*E* + 04	2.68*E* + 04
	Std.		2.50*E* + 03	1.43*E* + 03	1.37*E* + 03	1.30*E* + 03	5.75*E* + 02	6.74*E* + 03
	Time		4.32*E* + 00	4.35*E* + 00	4.34*E* + 00	7.42*E* + 00	3.97*E* + 00	2.24*E* + 00
	Rank		1	3	4	5	6	2
	Test	*p*	*—*	3.66*E *−* *13	6.42*E *−* *31	7.41*E *−* *30	7.94*E *−* *35	0.001689
		*h*	*—*	1	1	1	1	1

*F*23	Mean		1.51*E* + 03	2.80*E* + 03	2.98*E* + 03	4.25*E* + 03	4.52*E* + 03	2.25*E* + 03
	Std.		1.41*E* + 02	2.36*E* + 02	1.52*E* + 02	2.55*E* + 02	2.62*E* + 02	1.59*E* + 02
	Time		5.35*E* + 00	5.39*E* + 00	5.34*E* + 00	8.40*E* + 00	5.04*E* + 00	2.76*E* + 00
	Rank		1	3	4	5	6	2
	Test	*p*	*—*	1.58*E *−* *48	1.75*E *−* *71	1.63*E *−* *69	5.12*E *−* *71	1.50*E *−* *43
		*h*	*—*	1	1	1	1	1

*F*24	Mean		1.98*E* + 03	3.80*E* + 03	5.15*E* + 03	7.70*E* + 03	8.92*E* + 03	3.27*E* + 03
	Std.		1.76*E* + 02	3.91*E* + 02	3.10*E* + 02	3.21*E* + 02	6.13*E* + 02	2.66*E* + 02
	Time		5.64*E* + 00	5.69*E* + 00	5.61*E* + 00	8.76*E* + 00	5.37*E* + 00	2.90*E* + 00
	Rank		1	3	4	5	6	2
	Test	*p*	*—*	4.88*E *−* *41	1.93*E *−* *68	9.76*E *−* *86	4.72*E *−* *18	1.96*E *−* *45
		*h*	*—*	1	1	1	1	1
*F*25	Mean		2.18*E* + 03	4.32*E* + 03	2.16*E* + 04	1.89*E* + 04	2.73*E* + 04	5.76*E* + 03
	Std.		2.31*E* + 02	4.90*E* + 02	3.56*E* + 03	9.36*E* + 02	1.75*E* + 03	6.27*E* + 02
	Time		6.03*E* + 00	6.07*E* + 00	6.02*E* + 00	9.05*E* + 00	5.89*E* + 00	3.10*E* + 00
	Rank		1	2	5	4	6	3
	Test	*p*	*—*	1.17*E *−* *39	1.59*E *−* *38	8.88*E *−* *69	4.62*E *−* *60	1.34*E *−* *44
		*h*	*—*	1	1	1	1	1

*F*26	Mean		1.49*E* + 04	3.10*E* + 04	3.48*E* + 04	4.36*E* + 04	5.56*E* + 04	2.75*E* + 04
	Std.		1.76*E* + 03	3.10*E* + 03	4.04*E* + 03	1.60*E* + 03	1.47*E* + 03	3.09*E* + 03
	Time		6.65*E* + 00	6.69*E* + 00	6.63*E* + 00	9.73*E* + 00	6.28*E* + 00	3.42*E* + 00
	Rank		1	3	4	5	6	2
	Test	*p*	*—*	7.07*E *−* *18	7.07*E *−* *18	7.07*E *−* *18	7.07*E *−* *18	7.5*E *−* *18
		*h*	*—*	1	1	1	1	1

*F*27	mean		1.44*E* + 03	3.04*E* + 03	3.69*E* + 03	8.44*E* + 03	1.02*E* + 04	2.47*E* + 03
	std.		2.21*E* + 02	7.61*E* + 02	8.06*E* + 02	5.24*E* + 02	2.45*E* + 02	3.46*E* + 02
	Time		7.84*E* + 00	7.87*E* + 00	7.81*E* + 00	1.09*E* + 01	7.59*E* + 00	4.03*E* + 00
	Rank		1	3	4	5	6	2
	Test	*p*	*—*	1.95*E *−* *17	7.97*E *−* *18	7.07*E *−* *18	2.76*E *−* *19	2.78*E *−* *17
		*h*	*—*	1	1	1	1	1

*F*28	Mean		3.78*E* + 03	6.46*E* + 03	2.38*E* + 04	2.49*E* + 04	3.31*E* + 04	9.47*E* + 03
	Std.		9.04*E* + 02	6.23*E* + 02	3.18*E* + 03	3.05*E* + 03	1.07*E* + 03	1.19*E* + 03
	Time		7.34*E* + 00	7.36*E* + 00	7.38*E* + 00	1.03*E* + 01	7.12*E* + 00	3.76*E* + 00
	Rank		1	2	4	5	6	3
	Test	*p*	*—*	8.55*E *−* *30	5.55*E *−* *45	7.07*E *−* *18	4.56*E *−* *117	4.98*E *−* *47
		*h*	*—*	1	1	1	1	1

*F*29	Mean		7.47*E* + 03	1.28*E* + 04	1.89*E* + 04	4.87*E* + 04	7.55*E* + 05	1.01*E* + 04
	Std.		9.15*E* + 02	1.90*E* + 03	5.85*E* + 03	1.26*E* + 04	2.73*E* + 05	1.16*E* + 03
	Time		4.33*E* + 00	4.37*E* + 00	4.41*E* + 00	7.33*E* + 00	4.16*E* + 00	2.21*E* + 00
	Rank		1	3	4	5	6	2
	Test	*p*	*—*	5.82*E *−* *28	7.07*E *−* *18	4.38*E *−* *28	1.39*E *−* *24	5.84*E *−* *22
		*h*	*—*	1	1	1	1	1

*F*30	Mean		4.77*E* + 08	1.43*E* + 09	7.26*E* + 09	2.56*E* + 10	4.83*E* + 10	1.06*E* + 09
	Std.		1.99*E* + 08	5.49*E* + 08	2.28*E* + 09	3.75*E* + 09	2.84*E* + 09	3.97*E* + 08
	Time		6.96*E* + 00	7.01*E* + 00	7.01*E* + 00	9.93*E* + 00	6.54*E* + 00	3.57*E* + 00
	Rank		1	3	4	5	6	2
	Test	*p*	*—*	4.41*E *−* *17	2.49*E *−* *26	7.07*E *−* *18	7.07*E *−* *18	4.23*E *−* *14
		*h*	*—*	1	1	1	1	1

Average rank			1	2.8	4.1	4.9	6	2.2
Overall rank			1	3	4	5	6	2

**Table 15:** The statistical results of significance test.

Algorithm	*D*	Result numbers			
		*h* = 1+	*h* = 1−	*h* = 0+	*h* = 0−
GMO and WOA	10	27	0	1	1
	30	29	0	0	0
	50	29	0	0	0
	100	29	0	0	0

GMO and PSO	10	29	0	0	0
	30	29	0	0	0
	50	29	0	0	0
	100	29	0	0	0

GMO and BRO	10	26	2	0	1
	30	29	0	0	0
	50	29	0	0	0
	100	28	1	0	0

GMO and CSO	10	29	0	0	0
	30	29	0	0	0
	50	29	0	0	0
	100	28	0	0	1

GMO and ABC	10	20	2	7	0
	30	27	0	0	2
	50	26	2	0	1
	100	28	1	0	0

Proportion (%)		96.2	1.4	1.4	1.0

**Table 16:** The statistical results of running time.

*D*	Index	GMO		WOA		PSO		BRO		CSO		ABC	
		Time	*F*(*i*)	Time	*F*(*i*)	Time	*F*(*i*)	Time	*F*(*i*)	Time	*F*(*i*)	Time	*F*(*i*)
10	Best	0.182	*F*3	0.189	*F*1	1.148	*F*5	2.234	*F*1	0.275	*F*1	0.89	*F*3
	Median	0.216	*F*9	0.220	*F*15	0.180	*F*10	2.286	*F*12	0.302	*F*9	0.11	*F*15
	Worse	0.593	*F*30	0.595	*F*30	0.552	*F*30	2.662	*F*30	0.643	*F*30	0.299	*F*30
	Mean	0.258	—	0.263	—	0.224	—	2.258	—	0.224	—	0.128	—

30	Best	0.308	*F*15	0.321	*F*15	0.276	*F*15	2.622	*F*12	0.367	*F*15	0.139	*F*15
	Median	0.559	*F*14	0.581	*F*10	0.472	*F*14	3.785	*F*10	0.741	*F*18	0.341	*F*6
	Worse	1.666	*F*19	1.690	*F*19	1.582	*F*19	5.018	*F*19	1.766	*F*19	0.970	*F*19
	Mean	0.718	—	0.741	—	0.642	—	3.731	—	0.878	—	0.431	—

50	Best	0.412	*F*4	0.421	*F*4	0.385	*F*1	2.876	*F*4	0.490	*F*4	0.197	*F*4
	Median	0.601	*F*10	0.612	*F*10	0.567	*F*14	3.051	*F*10	0.605	*F*10	0.295	*F*14
	Worse	2.699	*F*30	2.710	*F*30	2.659	*F*30	5.224	*F*30	2.536	*F*30	1.381	*F*30
	Mean	0.966	—	0.979	—	0.935	—	3.357	—	0.967	—	0.486	—

100	Best	1.323	*F*1	1.360	*F*1	1.337	*F*1	4.251	*F*1	1.395	*F*1	0.654	*F*1
	Median	1.655	*F*14	1.686	*F*14	1.693	*F*14	4.624	*F*12	1.599	*F*14	0.823	*F*14
	Worse	7.84	*F*27	7.868	*F*27	7.810	*F*27	10.914	*F*27	7.589	*F*27	4.028	*F*27
	Mean	3.057	—	3.093	—	3.077	—	5.975	—	2.961	—	1.557	—

**Table 17:** Comparison with some studies in the literature.

Function	GMO		FDB-SOS		FDBSFS		FSA [59]		KABC	
	Mean	Std.	Mean	Std.	Mean	Std.	Mean	Std.	Mean	Std.
*F*1	2.96*E* + 04	2.13*E* + 04	1.19*E* + 07	5.64*E* + 06	1.31*E* + 07	1.61*E* + 07	5.70*E* + 03	7.68*E* + 03	8.99*E* + 03	1.42*E* + 04
*F*3	2.52*E* + 04	9.19*E* + 03	1.86*E* + 04	7.31*E* + 03	2.21*E* + 04	6.09*E* + 03	8.59*E* + 04	9.54*E* + 03	1.09*E* + 05	2.30*E* + 04
*F*4	1.17*E* + 02	1.96*E* + 01	1.96*E* + 02	5.32*E* + 01	1.26*E* + 02	3.62*E* + 01	1.18*E* + 02	9.77*E* + 01	4.80*E* + 02	3.50*E* + 01
*F*5	1.21*E* + 02	3.37*E* + 01	2.09*E* + 01	8.40*E* − 02	1.08*E* + 02	2.45*E* + 01	6.83*E* + 02	3.84*E* + 01	5.61*E* + 02	1.13*E* + 01
*F*6	3.07*E* + 01	9.70*E* + 00	3.92*E* + 01	4.91*E* + 00	1.52*E* + 00	8.07*E* − 01	6.43*E* + 02	1.35E-02	6.00*E* + 02	1.57*E* − 02
*F*7	1.79*E* + 02	3.81*E* + 01	4.84*E* − 01	2.17*E* − 01	1.65*E* + 02	3.05*E* + 01	8.09*E* + 02	2.34*E* + 01	8.02*E* + 02	1.88*E* + 01
*F*8	1.22*E* + 02	3.09*E* + 01	2.37*E* + 02	3.11*E* + 01	9.65*E* + 01	2.10*E* + 01	6.72*E* + 02	3.61*E* + 01	8.57*E* + 02	1.56*E* + 01
*F*9	1.36*E* + 03	7.41*E* + 02	2.98*E* + 02	7.62*E* + 01	2.70*E* + 02	2.54*E* + 02	1.03*E* + 01	9.71*E* + 02	1.05*E* + 03	1.50*E* + 02
*F*10	4.56*E* + 03	8.56*E* + 02	6.44*E* + 03	7.90*E* + 02	4.05*E* + 03	6.12*E* + 02	4.90*E* + 03	6.81*E* + 02	7.62*E* + 03	1.27*E* + 03
*F*11	2.14*E* + 02	6.42*E* + 01	9.14*E* + 03	1.46*E* + 03	1.40*E* + 02	4.41*E* + 01	1.16*E* + 03	5.22*E* + 02	1.16*E* + 03	2.88*E* + 01
*F*12	9.13*E* + 06	7.86*E* + 06	1.58*E* + 00	3.45*E* − 01	1.90*E* + 06	1.96*E* + 06	6.61*E* + 04	9.59*E* + 05	2.72*E* + 06	2.17*E* + 06
*F*13	1.68*E* + 05	7.11*E* + 04	6.21*E* − 01	1.04*E* − 01	2.01*E* + 04	2.57*E* + 04	1.46*E* + 04	1.02*E* + 03	2.01*E* + 04	2.21*E* + 04
*F*14	1.27*E* + 03	2.68*E* + 03	3.99*E* − 01	1.44*E* − 01	4.19*E* + 03	7.55*E* + 03	4.54*E* + 04	6.21*E* + 04	3.67*E* + 04	2.20*E* + 04
*F*15	4.74*E* + 04	3.30*E* + 04	8.34*E* + 01	2.20*E* + 01	4.31*E* + 03	3.54*E* + 03	1.84*E* + 04	8.19*E* + 04	7.35*E* + 03	7.82*E* + 03
*F*16	1.07*E* + 03	2.98*E* + 02	2.12*E* + 01	5.78*E* − 01	8.90*E* + 02	2.72*E* + 02	2.64*E* + 03	1.47*E* + 02	2.39*E* + 03	2.55*E* + 02
*F*17	4.00*E* + 02	1.94*E* + 02	1.72*E* + 06	1.28*E* + 06	2.69*E* + 02	1.63*E* + 02	2.56*E* + 03	5.48*E* + 02	1.95*E* + 03	1.25*E* + 02
*F*18	1.09*E* + 05	6.86*E* + 04	2.59*E* + 03	1.68*E* + 03	9.71*E* + 04	6.31*E* + 04	5.20*E* + 05	9.74*E* + 05	6.19*E* + 05	5.62*E* + 05
*F*19	1.77*E* + 05	2.08*E* + 05	6.48*E* + 01	3.26*E* + 01	3.35*E* + 03	4.82*E* + 03	5.22*E* + 03	1.29*E* + 04	1.17*E* + 04	1.23*E* + 04
*F*20	5.09*E* + 02	1.31*E* + 02	1.52*E* + 04	5.33*E* + 03	3.36*E* + 02	1.47*E* + 02	2.59*E* + 03	1.07*E* + 02	2.34*E* + 03	1.52*E* + 02
*F*21	3.31*E* + 02	2.92*E* + 01	6.75*E* + 05	3.74*E* + 05	2.80*E* + 02	4.55*E* + 01	2.21*E* + 03	1.19*E* + 02	2.36*E* + 03	1.42*E* + 01
*F*22	2.33*E* + 02	8.90*E* + 02	1.19*E* + 03	3.86*E* + 02	1.14*E* + 02	3.47*E* + 00	3.10*E* + 03	7.25*E* + 03	8.38*E* + 03	2.68*E* + 03
*F*23	4.77*E* + 02	4.03*E* + 01	2.00*E* + 02	0.00*E* + 00	4.60*E* + 02	2.44*E* + 01	2.59*E* + 03	1.12*E* + 02	2.72*E* + 03	2.44*E* + 01
*F*24	5.34*E* + 02	3.28*E* + 01	2.00*E* + 02	1.01*E* − 04	5.20*E* + 02	2.53*E* + 01	2.97*E* + 03	2.66*E* + 02	2.91*E* + 03	1.79*E* + 01
*F*25	4.28*E* + 02	2.59*E* + 01	2.00*E* + 02	0.00*E* + 00	4.14*E* + 02	1.70*E* + 01	2.78*E* + 03	9.32*E* + 00	2.89*E* + 03	9.44*E* + 00
*F*26	1.71*E* + 03	1.14*E* + 03	1.81*E* + 02	3.94*E* + 01	1.63*E* + 03	8.74*E* + 02	3.14*E* + 03	8.55*E* + 02	3.99*E* + 03	3.54*E* + 02
*F*27	5.65*E* + 02	2.54*E* + 01	1.37*E* + 03	1.24*E* + 02	5.54*E* + 02	1.98*E* + 01	2.95*E* + 03	9.86*E* + 01	3.20*E* + 03	1.65E-04
*F*28	5.04*E* + 02	2.78*E* + 01	2.36*E* + 03	8.66*E* + 02	4.78*E* + 02	3.63*E* + 01	3.36*E* + 03	5.14*E* + 01	3.30*E* + 03	4.73*E* + 00
*F*29	1.34*E* + 03	2.13*E* + 02	2.28*E* + 07	1.94*E* + 07	8.10*E* + 02	1.35*E* + 02	3.61*E* + 03	3.51*E* + 01	3.57*E* + 03	1.88*E* + 02
*F*30	1.89*E* + 06	1.46*E* + 06	1.52*E* + 04	6.56*E* + 03	6.33*E* + 04	8.39*E* + 04	3.40*E* + 03	4.13*E* + 02	6.43*E* + 03	2.84*E* + 03

**Table 18:** Comparison results for the three-bar truss design problem.

Algorithm	*x*_1_	*x*_2_	Optimum cost
GMO	0.7886775	0.4082415	263.8958434
KABC	0.7886	0.4084	263.8959
DMMFO	0.788687421	0.408213541	263.8958435
GOA	0.7888976	0.4076196	263.895881
ALO	0.788662816000317	0.408283133832901	263.8958434
CS	0.78867	0.40902	263.9716
GSA	0.7886751284	0.4082483080	263.8958434
MBA	0.7885650	0.4085597	263.8958522

**Table 19:** Comparison results for pressure vessel design problem.

Algorithm	*x*_1_	*x*_2_	*x*_3_	*x*_4_	Optimum cost
GMO	0.8125	0.4375	42.0984456	176.6365958	6059.7143
KABC	0.8745	0.4323	45.3106	140.5353	5951.593
DMMFO	0.7430	0.3842	40.319619	200.0000	6032.5484
LSA	0.8125	0.43750	42.097398	176.65405	6059.9463
CS	0.8125	0.4375	42.0984456	176.6365958	6059.7143
GSA	0.8125	0.437500	42.09844539	176.63659855	6059.7144
LAPSO	0.8125	0.4375	42.0984	176.6366	6059.7143
CPSO	0.8125	0.437500	42.091266	176.746500	6061.0777
MABGA	0.7917	0.3924	41.0218	190.4508	5912.2
MBA	0.7802	0.3856	40.4292	198.4964	5889.3216

**Table 20:** Comparison results for the tension/compression spring design problem.

Algorithm	*x*_1_	*x*_2_	*x*_3_	Optimum cost
GMO	0.0514617	0.3512730	11.6154626	0.0126662
KABC	0.0556	0.4575	7.1480	0.013017
LSA	0.05027598	0.32367954	13.52540953	0.012720452
CPSO	0.051728	0.357644	11.244543	0.0126747
MBA	0.051656	0.355940	11.344665	0.012665
CBO	0.051894	0.3616740	11.007846	0.0126697

**Table 21:** Comparison result of the gear train design problem.

Algorithm	*x*_1_	*x*_2_	*x*_3_	*x*_4_	Optimum cost
GMO	43	19	16	49	2.700857*E* − 12
KABC	50.4259	22.3987	16.7082	51.4394	0
ALO	43	19	16	49	2.7009*E* − 12
IAPSO	43	19	16	49	2.700857*E* − 12
MBA	43	19	16	49	2.700857*E* − 12

**Table 22:** Comparison results for the cantilever beam design problem.

Algorithm	*x*_1_	*x*_2_	*x*_3_	*x*_4_	*x*_5_	Optimum cost
GMO	6.0148311	5.3106005	4.4960591	3.5010936	2.1510803	1.3399567
GOA	6.011674	5.31297	4.48307	3.50279	2.16333	1.33996
ALO	6.01812	5.31142	4.48836	3.49751	2.158329	1.33995
CS	6.0089	5.3049	4.5023	3.5077	2.1504	1.33999
SOS	6.01878	5.30344	4.49587	3.49896	2.15564	1.33996

**Table 23:** *D-H* parameters of IRB5400 robot.

No	*a*_*i*_ (meters)	*d*_*i*_ (meters)	*α*_*i*_ (°)	*θ*_*i*_ (°)
1	0	0.66	0	*θ*_1_
2	0.3	0	−90	*θ*_2_
3	1.2	0	0	*θ*_3_
4	0.186	0.14075	−90	*θ*_4_
5	0	0.07935	35	−*θ*_6_
6	0	0.07935	−70	*θ*_6_
7	0	0.082501	35	*θ*_7_

**Table 24:** Experimental test points.

Test points	Robot end pose matrix (_7_^0^ *T*)
Point 1	$\left[\begin{array}{cc} \begin{array}{cc} -0.17931106457 & -0.08651059806\\ -0.27296478268 & -0.95263994316 \end{array} & \begin{array}{cc} -0.97998135622 & +1.31356028780\\ +0.13404240413 & +1.09112643623 \end{array}\\ \begin{array}{cc} -0.94516547204 & +0.29153568412\\ 0.00000000000 & 0.00000000000 \end{array} & \begin{array}{cc} +0.14720453576 & -0.09275569962\\ 0.00000000000 & +1.00000000000 \end{array} \end{array}\right]$
Point 2	$\left[\begin{array}{cc} \begin{array}{cc} +0.85283591032 & -0.06535445456\\ -0.01552116481 & -0.99487122809 \end{array} & \begin{array}{cc} -0.51807306950 & +1.72688939209\\ +0.09995165315 & +0.98941272857 \end{array}\\ \begin{array}{cc} -0.52194827667 & -0.07720126160\\ 0.00000000000 & 0.00000000000 \end{array} & \begin{array}{cc} -0.84947628671 & +0.97503807418\\ 0.00000000000 & +1.00000000000 \end{array} \end{array}\right]$

**Table 25:** Experimental results of 6 algorithms on two test points.

		GMO	WOA	PSO	BRO	CSO	ABC
Point 1	Mean	1.68*E* − 11	8.6–*E* − 02	2.8–*E* − 04	2.48–*E* − 02	3.0–*E* − 01	8.4–*E* − 05
	Best	6.1–*E* − 16	1.7–*E* − 02	5.5–*E* − 05	4.2–*E* − 03	1.2–*E* − 01	3.4–*E* − 05
	Worst	7.7–*E* − 10	2.7–*E* − 01	1.3–*E* − 03	1.0–*E* − 01	5.6–*E* − 01	1.7–*E* − 04
	Std.	1.1–*E* − 10	5.6–*E* − 02	2.1–*E* − 04	1.9–*E* − 02	8.7–*E* − 02	3.5–*E* − 05

Point 2	Mean	7.1–*E* − 12	6.2–*E* − 02	8.7–*E* − 05	2.72–*E* − 03	2.2–*E* − 01	6.3–*E* − 05
	Best	6.0–*E* − 16	3.8–*E* − 03	3.5–*E* − 05	1.6–*E* − 05	3.5–*E* − 02	1.5–*E* − 05
	Worst	3.1–*E* − 10	2.5–*E* − 01	1.8–*E* − 04	1.2–*E* − 02	5.0–*E* − 01	1.2–*E* − 04
	Std.	4.5–*E* − 11	5.6–*E* − 02	3.3–*E* − 05	2.6–*E* − 03	1.0–*E* − 01	2.2–*E* − 05

**Table 26:** Errors of elements in the pose matrix.

Test points	Index	GMO	WOA	PSO	BRO	CSO	ABC
Point 1	*n* − *n* ^∧^	2.78*E* − 17 3.33*E* − 16 2.22*E* − 16	−8.21*E* − 04 3.41*E* − 04 5.78*E* − 05	−1.93*E* − 05 4.74*E* − 06 2.29*E* − 06	2.59*E* − 04 −1.14*E* − 04 −1.63*E* − 05	2.23*E* − 02 −4.01*E* − 03 −2.80*E* − 03	6.93*E* − 06 −2.13*E* − 06 −7.00*E* − 07
	*o* − *o* ^∧^	1.11*E* − 16 2.22*E* − 160	−3.42*E* − 03 4.04*E* − 04 2.85*E* − 04	1.18*E* − 05 −2.26E*E* − 06 −3.89*E* − 06	−7.94*E* − 04 1.33*E* − 04 1.98*E* − 04	−3.14 *E* − 02 4.89 *E* − 03 4.90*E* − 03	−6.31*E* − 06 1.23*E* − 06 2.14*E* − 06
	*α* − *α* ^∧^	3.33*E* − 16 1.39*E* − 16 1.67*E* − 16	4.59*E* − 04 3.52*E* − 03 −1.94*E* − 04	2.49*E* − 06 −6.40*E* − 06 2.24*E* − 05	2.30*E* − 05 7.11*E* − 04 −4.98*E* − 04	−5.57*E* − 04 2.42*E* − 02 3.11*E* − 02	−7.11*E* − 07 4.39*E* − 06 −8.73*E* − 06
	*p* − *p* ^∧^	0 0 0	−6.79*E* − 03 7.55*E* − 03 −6.86*E* − 03	−1.76*E* − 05 6.43*E* − 06 −1.19*E* − 05	−2.09*E* − 04 2.61*E* − 03 1.51*E* − 03	4.57*E* − 02 4.22*E* − 02 1.40*E* − 02	−9.92*E* − 06 1.56*E* − 05 −8.23*E* − 06

Point 2	*n* − *n* ^∧^	−2.22*E* − 16 9.37*E* − 17 1.11*E* − 16	1.48 *E* − 04 1.00*E* − 03 2.13*E* − 04	5.74*E* − 06 −5.29*E* − 06 9.53*E* − 06	−2.44*E* − 06 −5.94*E* − 06 −3.82*E* − 06	3.50 *E* − 03 5.60 *E* − 03 5.70 *E* − 03	1.01*E* − 06 3.41*E* − 06 1.53*E* − 06
	*o* − *o* ^∧^	6.94*E* − 17 1.11*E* − 16 −2.50*E* − 16	4.98*E* − 04 5.95*E* − 05 −1.20*E* − 03	−3.59*E* − 06 7.33*E* − 08 2.09*E* − 06	−3.49*E* − 06 −2.75*E* − 07 6.50*E* − 06	7.00 *E* − 03 −4.03 *E* − 04 −4.01 *E* − 04	8.67*E* − 07 3.66*E* − 07 −5.45*E* − 06
	*α* − *α* ^∧^	−2.22*E* − 16 −2.08*E* − 16 0	1.81*E* − 04 7.42*E* − 04 −2.29*E* − 05	9.90*E* − 06 −9.15*E* − 08 −6.05*E* − 06	−3.58*E* − 06 −3.66*E* − 06 1.76*E* − 06	5.00 *E* − 03 −3.40 *E* − 03 −3.40 *E* − 03	1.54*E* − 06 4.17*E* − 06 −4.47*E* − 07
	*p* − *p* ^∧^	0 1.11*E* − 16 0	1.40*E* − 03 −2.27*E* − 04 1.40*E* − 03	−1.27*E* − 06 −6.25*E* − 06 1.70*E* − 05	−3.06*E* − 06 −6.02*E* − 07 −3.00*E* − 06	8.10 *E* − 03 6.60 *E* − 03 −1.98 *E* − 02	9.85*E* − 07 −1.70*E* − 06 7.00*E* − 06

**Table 27:** Comparison results of inverse kinematics solutions.

Algorithms	Results	Robot	IK problem
GMO algorithm in this paper	1.68*E* − 11	7R 6DOF	Pose error
Modified ABC [76]	6.31*E* − 13	6DOF	Position error
ANN [77]	0.001665	5 DOF	Position error
Quantum PSO [78]	2.775*E* − 17	7 DOF	Position error
Global-local best IW PSO [79]	3,64*E* − 03	7 DOF	Position error
Firefly [80]	6.53*E* − 05	7 DOF	Position error
Improves PSO [81]	4.00*E* − 04	6 DOF	Position error
CMA-ES [82]	0.1441	5 DOF	Position error
BRO [25]	1.8914*E* − 07	6 DOF	Position error
NIKA [83]	1.02*E* − 04	6 DOF	Position error
KABC [60]	1.62*E* − 04	5 DOF	Position error
NMFOA [84]	2.151*E* − 3	7 DOF	Pose error
IW-PSO [85]	6.655*E* − 05	2 DOF	Position error
SRM-PSO [86]	4.863*E* − 14	7 DOF	Position error

## Data Availability

The data used to support the findings of this study are included within the article, and the datasets and codes are available from the corresponding author on reasonable request.
